# Quantifying cell traction forces at the single-fiber scale in 3D: An approach based on deformable photopolymerized fiber arrays

**DOI:** 10.1073/pnas.2507677122

**Published:** 2025-10-13

**Authors:** Pierre Ucla, Joanne Lê-Chesnais, Henri Ver Hulst, Xingming Ju, Isabel Calvente, Elnaz Nematollahi, Ludovic Leconte, Jean Salamero, Isabelle Bonnet, Catherine Monnot, Hélène D. Moreau, Jessem Landoulsi, Vincent Semetey, Sylvie Coscoy

**Affiliations:** ^a^Institut Curie, Université PSL, Sorbonne Université, CNRS UMR168, Physics of Cells and Cancer, Paris 75005, France; ^b^Chimie ParisTech, PSL University, CNRS, Institut de Recherche de Chimie Paris, Paris 75005, France; ^c^Sorbonne Université, CNRS, Laboratoire de Réactivité de Surface, LRS, Paris F-75005, France; ^d^Institut Curie, PSL University, Inserm U932, Immunity and Cancer, Paris 75005, France; ^e^SERPICO/STED Team, UMR144 CNRS Institut Curie, Université PSL, Sorbonne Université, Paris 75005, France; ^f^Inria Centre Rennes-Bretagne Atlantique, Rennes cedex 35042, France; ^g^Centre de Recherche des Cordeliers, INSERM UMR_S 1138, Sorbonne Université, Université Paris Cité, Paris 75006, France

**Keywords:** 3D traction forces, two-photon polymerization, fibers, atomic force microscopy, cell contractility

## Abstract

The forces exerted by cells upon the fibers of the extracellular matrix play a decisive role in cell motility in physiopathology. How the local physical properties of the matrix (density, stiffness, orientation) affect cellular forces remains, however, poorly understood. Existing approaches to measure cell three-dimensional (3D) traction forces within fibrous substrates lack control over the local properties and rely on continuum approaches, not suited for measuring forces at the scale of individual fibers. Herein, an approach is proposed to fabricate multilayer arrays of suspended deformable fibers spanning a wide range of fine-tunable geometrical and mechanical properties using two-photon polymerization. Atomic Force Microscopy is used to thoroughly investigate the properties of individual fibers, including Young’s modulus and stiffness. This approach is combined with a reference-free method for measuring traction forces in 3D, which relies on automated segmentation of the fibers coupled with finite element modeling. The force measurement pipeline is applied to study forces exerted by endothelial cells, fibroblasts, or macrophages, and reveals how these forces are influenced by fiber density and stiffness. Additionally, coupling to fast volumetric imaging with lattice light-sheet microscopy enables the measurement of the low-intensity and short-lived tractions exerted by amoeboid cells, such as dendritic cells. Our technology will be instrumental for monitoring and studying cell behavior at the single-fiber level at extracellular matrix density interfaces, which play a crucial role in both physiological and pathological contexts, such as tumor boundaries.

## Significance

Cells evolve within intricate, fiber-rich microenvironments that present significant challenges for studying their behavior and mechanics. To address this, we employed advanced three-dimensional (3D) microfabrication to create parallelized arrays of fibers with controlled geometry and mechanics that can be deformed by cellular forces. This system is integrated with an automated pipeline to measure these forces in three dimensions. We validated the platform using various models, from connective tissue or blood vessel cells to immune cells, demonstrating its ability to analyze a wide range of cellular dynamics and adhesion forces. By replicating fibrillar environments with tunable architectures and mechanics, this approach provides a powerful tool for characterizing cell mechanics, offering valuable insights into cell interaction with complex, customizable mechanical landscapes.

Force generation is an essential feature of cell physiology that is important for a variety of processes such as migration, differentiation, and signaling. The mechanical and topographical cues provided by the network of fibrous proteins in the natural extracellular matrix (ECM) regulate cell adhesion, force generation, and, more broadly, mechanotransduction (1). The relationship between local physical properties—such as matrix density, stiffness, and fiber orientation—and force generation is especially critical since, in pathological conditions like cancer, the ECM is often remodeled, becoming highly heterogeneous (2). This leads to the generation of 3D gradients of stiffness and fiber density that strongly affect cell fate and behavior. Understanding how local physical properties influence force generation at the scale of individual collagen fibers is fundamental to decipher mechanisms of ECM remodeling, cell migration, and metastasis. However, measuring the forces generated by cells in situ (3) in their native context is challenging and offers little control over the properties of the local microenvironment. In past decades, a large array of tools has been developed to control mechanical properties and measure traction forces on two-dimensional (2D) substrates in vitro, but it has become increasingly evident that mechanobiology studies must address forces in 3D (4).

Hydrogels commonly used to study cell mechanobiology in 3D in vitro include collagen or fibrin. The traction forces exerted by cells within these hydrogels are usually characterized using traction force microscopy (TFM), which measures the displacement of embedded fluorescent beads (5–9), although it is also possible to measure the displacement field of the fibers themselves using confocal reflectance microscopy (10).

Despite these developments, important limitations remain in accurately measuring the traction forces generated by cells in 3D hydrogel matrices. First, measurements from cells embedded within collagen- or fibrin-derived hydrogels may lack reproducibility due to the heterogeneous nature of these materials and their degradation by proteases secreted by the cells. Second, with TFM, the displacement field is calculated by comparing the imaged volume with a stress-free reference state acquired at the end of the experiment; any plastic deformations of the matrix during the experiment can result in an altered reference state and errors in force reconstruction. Third, the hydrogels are typically treated as homogeneous materials and characterized mechanically using large-scale rheology techniques, which does not fully account for the local heterogeneities in properties such as stiffness, pore size, or ligand density that cells sense at the scale of individual fibers (11–13). Engineered hydrogels (14) made from PEG (15) or alginate (16, 17) can help fine-tune these parameters, but they have a porous rather than fibrillar architecture. Finally, collagen- or fibrin-derived hydrogels display strong nonlinear features due to fiber buckling, straightening, or stretching. Material models that consider these features rely on continuum approaches with large representative volume elements which are not suited for measuring forces exerted on individual fibers (7).

Alternative approaches to produce simplified 3D structures using microfabrication techniques allow for increased control of the local physical properties of the substrate. Yet, their use in cell traction force measurement has largely been limited so far to 2D force measurements. The deflection of arrays of polydimethylsiloxane micropillars produced by stereolithography (18) can be used for precise force measurement and fine control of the rigidity experienced by cells (19). Yet, this approach only allows for the measurement of tangential forces and does not account for vertical forces. Alternatively, arrays of polystyrene fibers with submicron diameters, fabricated using the spinneret-based tunable engineered parameters technique, provide a robust framework for fiber micromechanics (20, 21), but this approach is also limited to the measurement of in-plane traction forces (22).

In contrast to the microfabrication methods described above, two-photon polymerization (TPP) allows tremendous freedom in the geometry, chemistry, and mechanical properties of 3D microstructures being fabricated. This technique has been used to produce a variety of 3D microscaffolds in order to study cell migration, morphology, and forces in response to architectural cues of the microenvironment (23–25). However, the range of forces measurable was largely limited by the high structural stiffness of these scaffolds and measurements have been so far restricted to in-plane (2D) forces of cells with high contractility (26).

In this paper, we propose a method for fabricating multilayer arrays of highly deformable hydrogel-based fibers with fully controlled geometry and mechanical properties and we combine it with an original pipeline to measure the 3D traction forces locally exerted by cells on these fibers. The range of sizes and mechanical properties of the produced fibers is characterized by Atomic Force Microscopy (AFM) both in imaging and force spectroscopy modes. The in-plane and out-of-plane deflections of these fibers submitted to cell traction forces are quantified using fast confocal fluorescence microscopy combined with an automated 3D image analysis framework. The traction forces are then calculated using a regularized inverse method based on finite element analysis. A key advantage of this method is that it does not require a reference to a stress-free state at the end of the experiment. This approach provides a versatile tool for life scientists to quantify cell contractility and reconstruct 3D traction force maps of cells confined within a fibrillary microscaffold with reproducible geometrical and mechanical properties. We validate the approach using it to quantify the traction forces of two well-characterized adherent cell types, NIH/3T3 fibroblasts and human umbilical vein endothelial cells (HUVEC), and illustrate how the exerted forces depend on fiber stiffness and density. Furthermore, we demonstrate the utility of our method for studying the low-amplitude forces characteristic of immune cells by quantifying the traction forces generated by murine macrophages. Additionally, we show that it can be easily adapted for use with fast volumetric imaging systems, such as lattice light-sheet microscopy, enabling high spatiotemporal resolution in 3D. We apply this approach to quantify the short-lived traction forces exerted by amoeboid cells, such as murine dendritic cells, and highlight the transient anchors and short-lived protrusions that contact and deflect the fibers.

## Results

### Microscaffold Design.

**Fig. 1.**
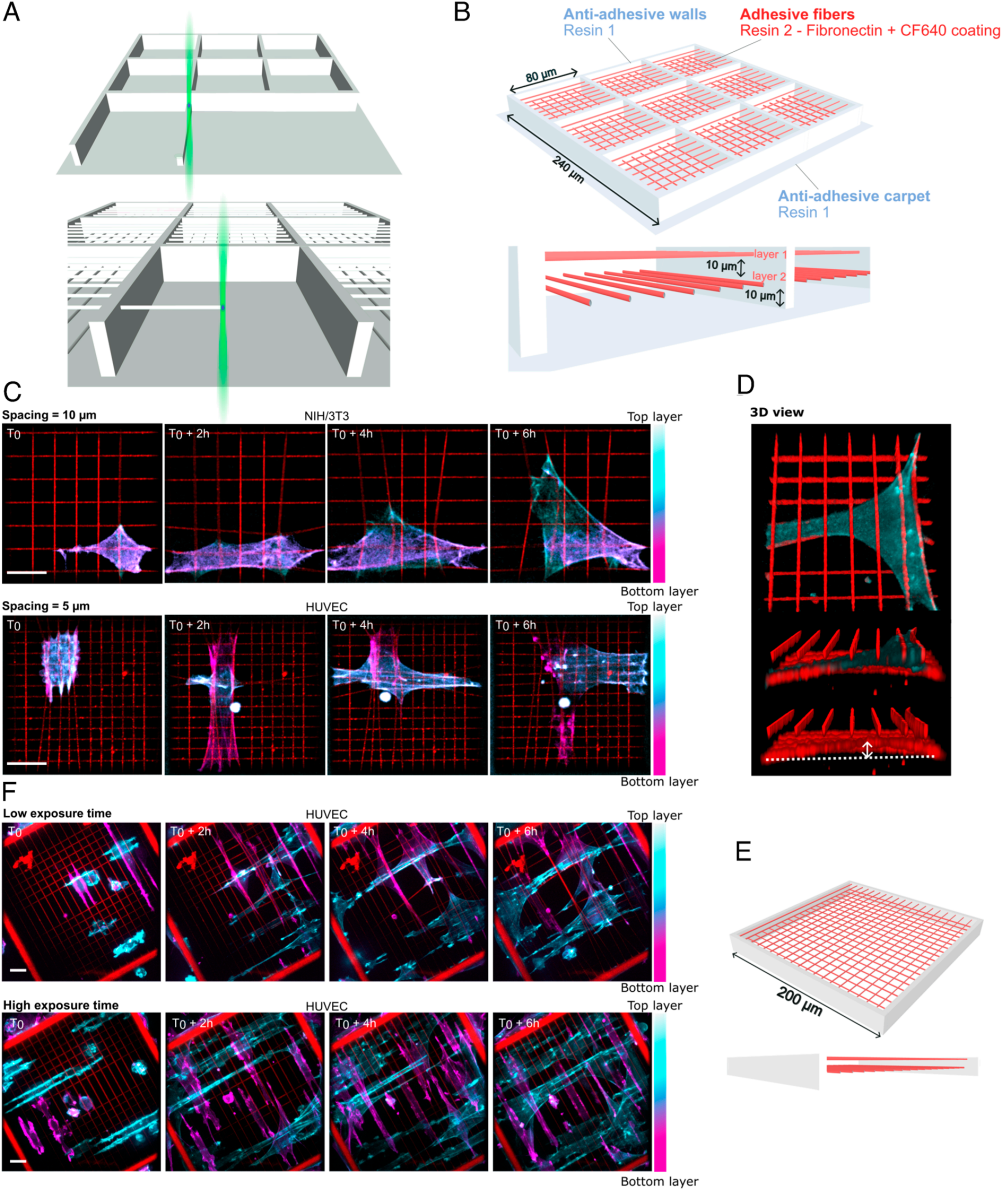
Photopolymerization of multilayered microscaffolds with fibers. (*A*) Two-step photopolymerization process. The structural parts are fabricated first with the antiadhesive resin (*Top*) before photopolymerization of the fibers as a single voxel line with the adhesive resin (*Bottom*). (*B*) Schematic of a 2-layer composite microscaffold with 80 μm fibers after the fabrication and fibronectin coating. *Top* view (*Top*) and *Side* view (*Bottom*). (*C*) Timelapse of a single NIH/3T3 Lifeact-GFP cell spreading and exerting traction forces on fibers with 10 μm lateral spacing (*Top*) and a HUVEC Lifeact-GFP cell on fibers with 5 μm lateral spacing (*Bottom*). (color scale: actin, red: fibers; maximum projection). (Scale bar: 20 μm.) (*D*) 3D reconstruction of an array with a single HUVEC Lifeact-GFP cell, with *Top* view (*Top*), *Side* view (*Middle*), and *Side* view of fibers only (*Bottom*). Vertical deflection of the fibers compared to their stress-free state is shown with a white arrow and dotted line. (*E*) Schematic of a 2-layer composite microscaffold with 200 μm-long fibers. (*F*) Timelapse of multiple HUVEC Lifeact-GFP cells spreading and exerting traction forces on highly deformable low exposure time photopolymerized fibers (*Top*) and on less deformable high exposure time photopolymerized fibers (*Bottom*). (color scale: actin, red: fibers; maximum projection). (Scale bar: 20 μm.)

Two-photon polymerization offers great flexibility for building 3D scaffolds with versatile geometries (27). We extended the range of this technique to create multilayer arrays of long deformable fibers by developing experimental procedures, including the careful selection of architecture design, materials, and processing methods. For simplicity, we focused on a two-layer fiber configuration, with suspended fibers printed orthogonally to maximize the freedom of conformations and spatial exploration of cells subjected to contact guidance on each layer (Fig. 1 *A*–*C*). Adding extra layers to the microscaffolds posed no technical challenges during the printing process (*SI Appendix*, Fig. S1). The fiber spacing was tunable in all dimensions, with a lower limit of 5 μm, set by fiber entanglement during fabrication and development. Here, we compared fiber spacings of 10 μm (Fig. 1 *C*, *Top*, Fig. 1 *D*–*F*) or 5 μm (Fig. 1 *C*, *Bottom*) in the *x-y* plane. In the *z* direction, a spacing of 10 μm was selected, small enough to allow cells to form protrusions between layers, facilitating 3D spreading of cells (Fig. 1*B*). In terms of materials, we designed composite microscaffolds composed of cell-adhesive fibers supported by cell-repellent structural part (walls and carpet) (Fig. 1 *A* and *B*). Resin 1, used for the structural parts, was composed of a mix of Polyethylene Glycol Diacrylate (PEGDA575) with 15% Pentaerythritol tetraacrylate (PETA), making it antiadhesive, while resin 2, used for the fibers, consisted of PEGDA250 with 10% PETA, allowing protein adsorption (28) (*SI Appendix*, Fig. S2). The fibers were then coated with fibronectin coupled with the highly photostable far-red dye CF™ 640R. We seeded NIH/3T3 Lifeact-GFP (Green Fluorescent Protein) fibroblasts and HUVEC Lifeact-GFP endothelial cells onto these microscaffolds with high cell viability 24 h after seeding (*SI Appendix*, Fig. S3). These two cell types were selected because both NIH/3T3 (5, 29–33) and HUVECs (34–37) have been widely used in traction force studies employing diverse experimental approaches, making them a reliable benchmark for validating our system. We confirmed that our 3D scaffold geometry favored cell adhesion, spreading, traction force exertion in the horizontal and vertical plane as well as migration of these adherent cells on the suspended fibers, with minimal cell adhesion to antiadhesive structural parts (Fig. 1 *C*–*F* and Movies S1 and S2).

### Geometrical and Mechanical Characterization of the Fibers.

A key aspect of our method is the generation of highly deformable fibers with controlled mechanical properties. These properties can be modulated by two fabrication parameters, laser power and scanning speed, with the latter controlling voxel exposure time. We observed that fiber deformability under cellular forces significantly decreased as exposure time increased, as demonstrated by the collective behavior of HUVECs on 200 μm grids (Fig. 1 *E* and *F* and Movies S3 and S4). This finding suggests that fiber stiffness can be fine-tuned by adjusting voxel exposure time. To build our force reconstruction model, we systematically examined the geometrical and mechanical properties of the photopolymerized fibers across different exposure times.

**Fig. 2.**
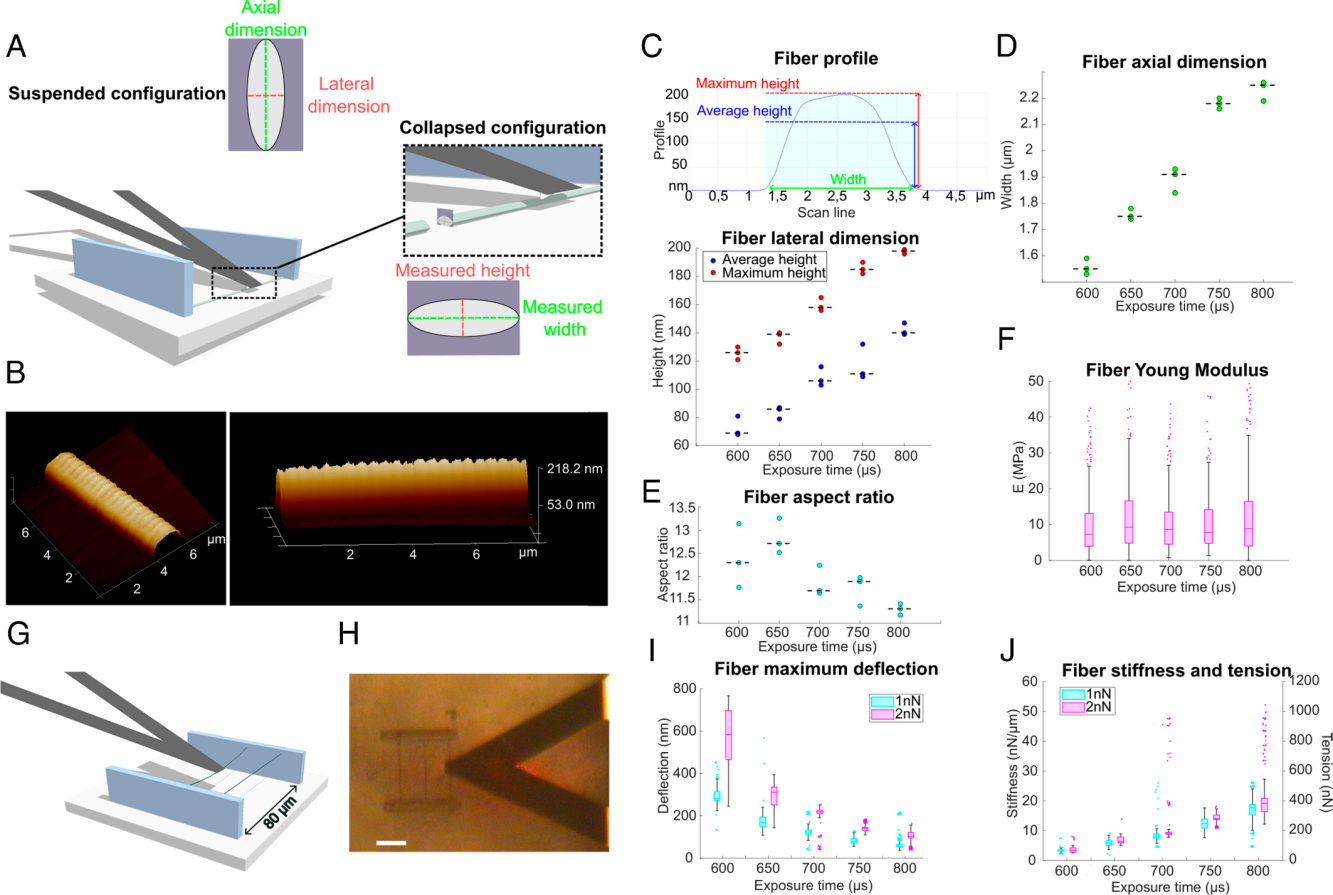
Geometrical and mechanical characterization of the photopolymerized fibers. (*A*) Scheme of the AFM topography and indentation measurements, illustrating how the AFM tip is brought in contact with the fiber that is collapsed on the glass coverslip. The fiber has an elliptic cross-section and rotates around its long axis when collapsing. (*B*) 3D view of a fiber for T_exp_ = 800 μs (*Left*: *Top* view, *Right*: *Side* view). (*C*) (*Top*) Scanning profile of a fiber for T_exp_ = 800 μs as measured by AFM. The average height corresponds to the mean value of the height profile function over the fiber interval. (*Bottom*) Variation of the lateral dimension of the produced fibers for a range of exposure times (N = 3 fibers for each condition). (*D*) Variation of the axial dimension of the produced fibers for a range of exposure times (N = 3 fibers for each condition). (*E*) Aspect ratio between axial dimension and maximum lateral dimension of the fibers. For all scatter plots, the dashed line indicates the median. (*F*) Young’s Modulus of the produced fibers for a range of exposure times as measured by AFM indentation (N = 3 fibers and n > 280 analyzed force curves for each condition). (*G*) Scheme and (*H*) bright field image of the suspended fiber deflection experiment, illustrating how the AFM tipless cantilever is brought in contact with the middle point of the suspended fiber. (Scale bar: 20 μm.) (*I*) Maximum deflection of the fiber, (*J*) corresponding stiffness and tension for an applied force of 1 nN and 2 nN (N = 3 fibers and n = 768 analyzed force-distance curves for each condition). For all box plots, the central mark indicates the median, the edges of the box denote the 25th and 75th percentiles, the whiskers extend to the most extreme data points not considered outliers, and outliers are plotted individually (dots).

First, we used AFM imaging to systematically measure the dimensions of fibers produced at a constant laser power (*Materials and Methods*), with exposure times ranging from 600 to 800 μs (*SI Appendix*, Fig. S4; see *SI Appendix*, Table S1 for the corresponding scanning speeds). Following the procedure described by Buchroithner et al. (38), fibers were collapsed on the substrate prior to AFM imaging. During this process, the fibers rotated along their axis, allowing measurement of the axial and lateral dimensions as the width and height of the collapsed fibers (Fig. 2 *A* and *B*). As expected for photopolymerized structures, the fiber cross-section was elliptical due to diffraction constraints and waveguide effect (39, 40) and the cross-section area increased with increasing exposure times. To quantify the lateral dimension of fibers, we measured both the maximum height and the average height (Fig. 2*C*), the latter being used to approximate a rectangular cross-section in our mechanical model. The maximum height increased from 126 ± 5 nm (mean ± SD) at 600 μs to 198 nm ± 2 nm at 800 μs and the average height increased from 73 ± 7 nm to 142 ± 4 nm. The axial dimension increased from 1.56 ± 0.03 μm at 600 μs to 2.33 ± 0.04 μm at 800 μs (Fig. 2*D*). The corresponding aspect ratios were relatively high and ranged between 11 and 13, with high aspect ratios already being reported in other studies (38, 41) (Fig. 2*E*). We also observed a periodic nanostructuration on the photopolymerized fibers, with an amplitude and a period decreasing as exposure time increased (*SI Appendix*, Fig. S5). Similar variations in photopolymerized voxel dimensions have been reported, likely linked to piezo oscillations affecting scanning speed (42). Next, nanomechanical indentation measurements using force spectroscopy were performed to determine the Young’s modulus of the collapsed fibers (*Materials and Methods* and *SI Appendix*, Fig. S6). The mean value was about 10 MPa (Fig. 2*F*, mean E = 11.32 ± 10.59 MPa, pooling all fiber conditions for a 1 nN load) consistent with previous mechanical characterization of PEGDA and PETA (38, 43, 44). The dispersion in Young’s modulus measurements was attributed to crosslinking differences between the core and the sides of fibers, as revealed by high-resolution mechanical mapping (*SI Appendix*, Fig. S7). As demonstrated in the following section, these variations only have a very limited impact on the overall mechanical behavior of the fibers, which appears to be primarily governed by built-in prestress, with only a minor contribution from the material’s intrinsic elasticity.

Across all tested exposure times, we observed shrinkage-induced tension accumulation during the development process with the fibers transitioning from a loose state in the monomer solution to a tense state after washing the uncured monomer with pure ethanol (Movie S5). At this stage, fibers can be described as clamped beams under tension. Their apparent stiffness at the midpoint results from the contribution of two components: structural stiffness, which is related to the elasticity of the material and the beam geometry, and stiffness resulting from the tensile loading. When structural elasticity dominates (that is when $\frac{\mathrm{EI}}{T}\gg 1)$, the apparent stiffness is given by $k_{\mathrm{app}}=k_{\mathrm{struct}}=\frac{192EI}{L^{3}}$. However, when tension dominates (i.e., when $\frac{\mathrm{EI}}{T}\ll 1)$, the apparent stiffness becomes $k_{\mathrm{app}}=k_{\mathrm{tension}}=\frac{4T}{L}$ (*SI Appendix*, *Supplementary Note 1*). Here, E is the Young’s modulus of the material, I is the fiber moment of inertia, L is the fiber length, and T is the tension.

In order to estimate the tension accumulated within the fibers during the development step, we measured the apparent stiffness of suspended fibers by performing force-distance measurements using an AFM tipless cantilever, brought into contact with the midpoint of each fiber (Fig. 2 *G* and *H*). We performed measurements with applied forces of 1 nN and 2 nN and recorded the resulting deflection (*SI Appendix*, Fig. S8). The deflection decreased as exposure time increased, confirming our ability to tune the fiber stiffness by adjusting fabrication parameters (Fig. 2 *I* and *J*). The corresponding apparent stiffness for a 1 nN force ranged from 3.5 ± 0.4 nN/μm (mean ± SD) at T_exp_ = 600 μs (3.6 ± 0.7 nN/μm for a 2 nN indentation force) to 17.6 ± 3.6 nN/μm at T_exp_ = 800 μs (respectively 19.8 ± 5.2 nN/μm). This spectrum matches the lower bounds described for pillar arrays (45) or STEP-deposited fibers (22) and is suitable for measuring a wide range of cell-generated forces. For clamped fibers, the apparent stiffness measured corresponds to the midpoint of the fiber, with the stiffness profile increasing nonlinearly along the fiber length (*SI Appendix*, Fig. S9). Based on the measured Young’s modulus and fiber dimensions, we estimated that the structural stiffness remained below 0.6 nN/μm for all exposure times studied, accounting for less than 3% of the total apparent stiffness. This indicates that the mechanical regime is largely dominated by tension. Using the average apparent stiffness measured for 1 nN and 2 nN applied forces, we calculated tension values for each condition, which ranged from 71 ± 12 nN for T_exp_ = 600 μs and 374 ± 93 nN for T_exp_ = 800 μs (Fig. 2*J*). The consistency between our mechanical model, measured fiber parameters, and fiber deflection under higher forces and different force application points is further detailed in *SI Appendix*, *Supplementary Note 4*. The geometrical and mechanical information extracted from the ensemble of AFM measurements was integrated to a pipeline enabling force reconstruction from fiber deflection measurements.

### A Constrained Reference-Free Inverse Traction Recovery Framework.

**Fig. 3.**
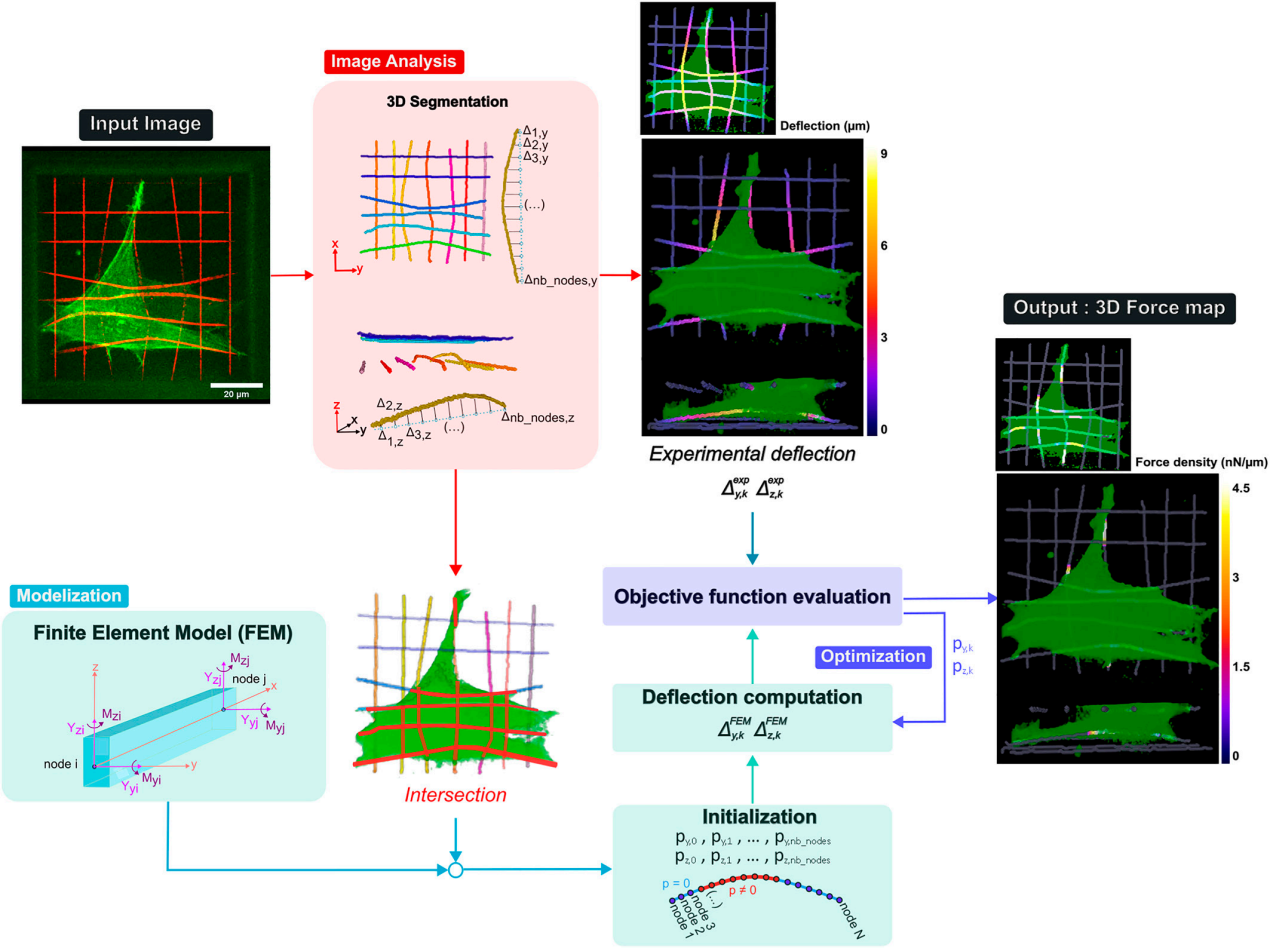
3D Traction forces recovery workflow. The input image stack is first treated by performing the 3D segmentation of the fibers. For each individual fiber, a deflection vector is computed in the two directions orthogonal to the axis of the fiber. The corresponding deflection map is generated where each voxel represents the sum of these two components. The cell volume is also segmented and the intersection between the cell and the fibers is computed to spatially constrain the subsequent traction force recovery. The force vector of a FEM based on 3D beam elements is initialized randomly in the intersection area and an optimization step is performed to minimize the distance between experimental and computed deflections. The output is a force vector for each fiber that is translated into a 3D force map.

We developed a Matlab workflow to compute fiber deflection (Fig. 3 and *SI Appendix*, *Supplementary Note 5*). Each individual fiber was segmented in 3D based on its fluorescent labeling and tracked over time (*SI Appendix*, *SI Materials and Methods*). A deflection vector was calculated as the distance between the deflected fiber skeleton and the straight line connecting its two anchored extremities. This approach offers the advantage of being reference-free, eliminating the need for cell trypsinization or the use of Cytochalasin D post-experiment to recover the stress-free state. The theoretical resolution of this deflection measurement is limited by the voxel size and could be lowered to the diffraction limit. However, experimental factors—such as imaging system resolution, acquisition frequency, cell sensitivity to phototoxicity or desired computation time—can affect this resolution. Under our experimental conditions, the resolution was 0.3 μm in the *x-y* plane and 1 μm in the *z* direction. These deflection measurements enabled the computation of a dynamic 3D deflection map, showing the local deflection of each fiber within the microscaffold at each time point, and used as the input of an inverse traction force recovery algorithm.

Finite Element Modeling (FEM) is traditionally used in 2D and 3D TFM to reconstruct the traction force field exerted on a bulk hydrogel, modeled as a discretized semi-infinite half space. In our case, FEM was used to study the mechanics of individual fibers. Each fiber was treated as a rectangular beam under tensile loading with clamped edges. It was divided into a finite number of 3D beam elements, each having 4 degrees of freedom: two transverse deflections (along y and z axes) and two rotations (around the *y* and *z* axes). Axial extension (in the direction of the fiber axis) and torsion were not considered (*SI Appendix*, *SI Materials and Methods* and *Supplementary Note 1* for details about the FE model). Ignoring axial forces leads to an underestimation of contractility which we evaluated by looking at the residual forces (*SI Appendix*, *Supplementary Note 3*). The inability of our system to resolve axial components represents a significant limitation in accurately interpreting cellular traction patterns in biological situations where axial forces predominate (*Discussion*).

The inverse problem of linking fiber deflection to the exerted forces was solved using an optimization framework. The force reconstruction was constrained to the area of intersection between the cell and the fibers. To avoid overfitting the model parameters, we used an elastic net (L1 + L2) regularization approach (46). The resulting loss function was as follows:

$$\begin{array}{c} \text{Loss}(p)=\sum_{k=0}^{\text{Nb}\_\mathrm{nodes}}\Delta_{\text{segmentation}\;y,k}-\Delta_{\text{model}\;y,k}\\ \;+\left|\Delta_{\mathrm{segmentation}\;z,k}-\Delta_{\text{model}\;z,k}\right|+\lambda_{1}||p||+\lambda_{2}||p|{|}^{2}, \end{array}$$

where **p** is the force vector, Δ_segmentation y,k_ and Δ_segmentation z,k_ are respectively the *y* and *z* deflection at node *k* extracted from experimental data, Δ_model y,k_ and Δ_model y,k_ are the deflections computed by the model, and λ_1_ and λ_2_ are the regularization parameters. The optimal regularization parameters were determined based on simulations, by generating artificial traction force patterns and introducing characteristic measurement noise into the corresponding deflection field (*SI Appendix*, *Supplementary Note 2*).

### Applications: 3D Traction Forces Maps and Cell Contractility Measurements.

**Fig. 4.**
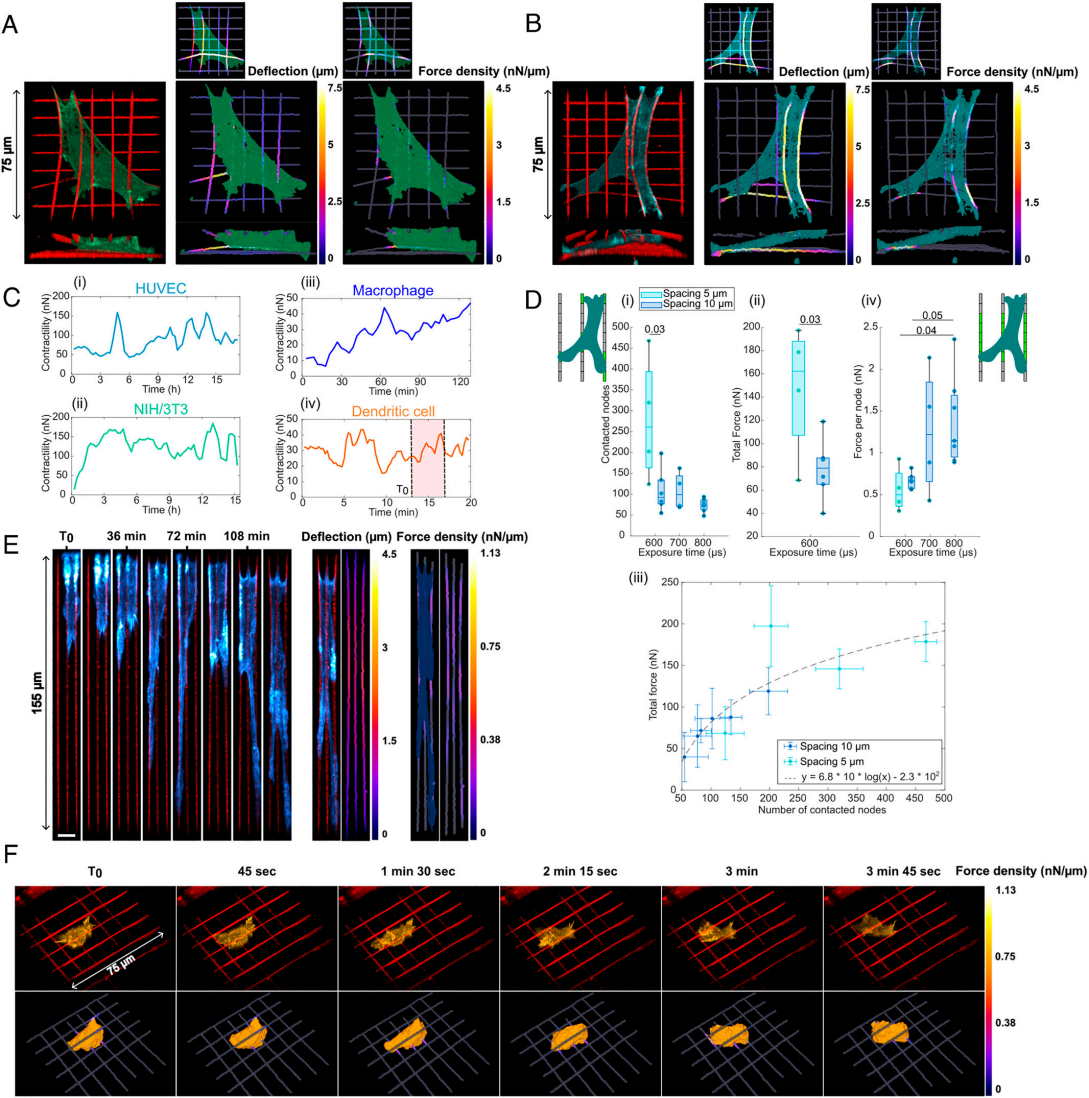
Examples of traction force profiles for various cell lines. 3D reconstruction (*Left*) of a NIH/3T3 cell (*A*) and a HUVEC (*B*) on a 2-layer fiber array with 10 μm lateral spacing and low-stiffness fibers (T_exp_ = 600 μs, k = 3.5 nN/μm) with the corresponding 3D deflection maps (*Center*) and 3D traction force maps (*Right*). Side views are represented on the *Bottom*. (*C*) Evolution of cell contractility over time for the respective examples of (*i*) HUVEC, (*ii*) NIH/3T3, (*iii*) macrophage and (*iv*) dendritic cell. (*D*) Effect of fiber exposure time and lateral spacing on (*i*) average number of contacted nodes, (*ii*) average total force and (*iv*) average force per node in the central part of the fiber for HUVECs. Measurements of individual cells are shown as points (600 μs, spacing 5 μm: N = 4; 600 μs, spacing 10 μm: N = 6; 700 μs: N = 4; 800 μs: N = 7). The two-sided *t* test was used for (*i*) and (*ii*) and Kruskal–Wallis test followed by multiple comparisons tests between groups using Dunn–Šidák correction was used for (*iv*). *P*-values are indicated. For all box plots, the central mark indicates the median, the edges of the box denote the 25th and 75th percentiles, the whiskers extend to the most extreme data points not considered outliers. (*iii*) Total force as a function of the number of contacted nodes and logarithmic fit (R^2^ = 0.76) for HUVECs on low-stiffness fibers (T_exp_ = 600 μs, k = 3.5 nN/μm) with lateral spacings of 5 μm (cyan) and 10 μm (blue). Each point represents the mean value over the whole timelapse for an individual cell and error bars correspond to the SD. (*E*) Timelapse illustrating adhesion and spreading of a macrophage Lifeact-GFP on one layer of medium-stiffness fibers (T_exp_ = 700 μs, k = 4.1 nN/μm) with lateral spacing of 5 μm while deflecting the fibers (*Left*) (blue: actin, red: fibers). (Scale bar: 10 μm.) Deflection map for one timepoint (*Center*) and corresponding force map with segmented cell mask (*Right*). (*F*) Lattice light-sheet timelapse of a dendritic cell migrating on a 2-layer fiber array with 10 μm lateral spacing and low-stiffness fibers (T_exp_ = 600 μs, k = 3.5 nN/μm) (*Top*) (orange: actin, red: fibers) and corresponding 3D traction force map (*Bottom*).

Traction forces were first investigated in two classical models of adherent cells known for generating high levels of contractile forces: NIH/3T3 fibroblasts and HUVEC endothelial cells (Fig. 4 *A* and *B* and Movies S6–S12). These cells spread, occasionally forming very long protrusions around fibers, as previously reported in other fiber-based bioengineered systems (47, 48). Cells contacted both *z* layers with possible transitions between fully spreading across both layers and predominantly interacting with one layer (Movies S6–S12). Traction force maps showed a spatial distribution of forces, with high force density at the cell periphery and protruding tips and weaker forces in the central region of the cell. Similar patterns of cellular tractions were previously reported on patterned hydrogels (49), micropost arrays (30), and suspended fibers (22), and theoretical models suggested that local traction forces depend linearly on the distance from the cell center (50). Contractility, measured as the sum of the exerted forces, typically ranged between 50 nN and 100 nN for HUVECs and 100 to 150 nN for NIH/3T3 with a 10 μm lateral spacing (Fig. 4 *C*, *i* and *ii*). Fig. 4 *C*, *ii* illustrates the characteristic increase in contractility associated with the spreading of a NIH/3T3 fibroblast over 2 to 3 h postseeding (Movie S6).

We sought to evaluate the ability of our system to modulate cell mechanics based on the local topography and mechanical properties of the fiber arrays. Specifically, we examined the influence of fiber density and stiffness on adhesion and traction forces generated by HUVECs. Cells were seeded on low-, middle-, or high-stiffness fibers with a lateral spacing of either 5 or 10 μm. We quantified cell adhesion by measuring the number of contacted nodes on the discretized fibers. On fibers with 5 μm lateral spacing, cells contacted more than twice the number of nodes (average of 279) compared with 10 μm fiber spacing (average of 108) (Fig. 4 *D*, *i*). For low-stiffness fibers, we observed a significant increase in total exerted force with fiber density, from 78 ± 26 nN (mean ± SD) for 10 μm spacing to 147 ± 57 nN for 5 μm spacing (Fig. 4 *D*, *ii*). This was associated with a logarithmic relationship between the total exerted force and the number of contacted nodes (Fig. 4 *D*, *iii*). Accordingly, the average force per node decreased with increasing number of contacted nodes (*SI Appendix*, Fig. S10), in agreement with previous studies on the effect of micropost density (30). Last, we examined the influence of fiber stiffness on the generated forces. We observed that the average force per node in the central area of the fiber (*SI Appendix*, Fig. S9) increased with fiber stiffness from 0.67 ± 0.10 nN/node to 1.4 ± 0.54 nN/node, respectively, for low- and high-stiffness fibers with a 10 μm spacing (Fig. 4 *D*, *iv*). This suggests that similar force–stiffness relationships govern traction forces in both 3D fibrillary systems and in simpler 2D systems (22, 51, 52). It is important to note, however, that the increase in fiber cross-section area with exposure time could also contribute to this increase of local forces. Altogether, these results demonstrate that both fiber density and stiffness modulate cell traction forces, influencing not only local forces but also the larger-scale mechanics across the entire fiber array.

To assess the potential of our method for measuring the lower range of traction forces typically generated by immune cells, we seeded macrophages Lifeact-GFP on very low-stiffness fibers, reducing their apparent stiffness by doubling the fiber length from 80 to 160 μm. Macrophages predominantly remained on the upper layer of the scaffolds. They adopted a highly elongated morphology with dynamic protrusions and contacted only two to three fibers at most, for the two lateral spacing considered (5 and 10 μm) (Fig. 4*E*) (Movies S13–S16). Measurable deflections in the range of 1 to 2 μm were observed, with measured contractility values between 10 nN and 50 nN (Fig. 4 *C*, *iii*), consistent with reported traction forces for leukocytes (9). Finally, 3D force measurements of extremely dynamic cell types remain challenging and require good compatibility with ultrafast volumetric imaging methods. To demonstrate the suitability of our setup, we combined it with Lattice Light-Sheet imaging to study the migration and force generation of dendritic cells on low-stiffness fibers. Although the motility of dendritic cell is classically described as amoeboid and adhesion independent (53, 54), we observed transient anchoring of the cells on the fiber via their uropod, and the formation of short-lived protrusions contacting and deflecting the fibers (Movies S17 and S18) (Fig. 4*F*). In contrast to macrophages, dendritic cells maintained a more rounded morphology and could frequently contact both layers of the scaffold. We measured a contractility in the range of 10 to 40 nN, consistent with previous studies on micropost arrays (55, 56), although slightly higher than what was measured on other suspended fiber systems (57), likely due to higher fiber density of our multilayer system. The compact shape of DCs resulted in higher local force density than for macrophages, and they also exhibited quick contractility variations, in the range of 1 to 5 min, similar to what was observed for natural killer cells in reconstituted collagen matrices (58).

**Fig. 5.**
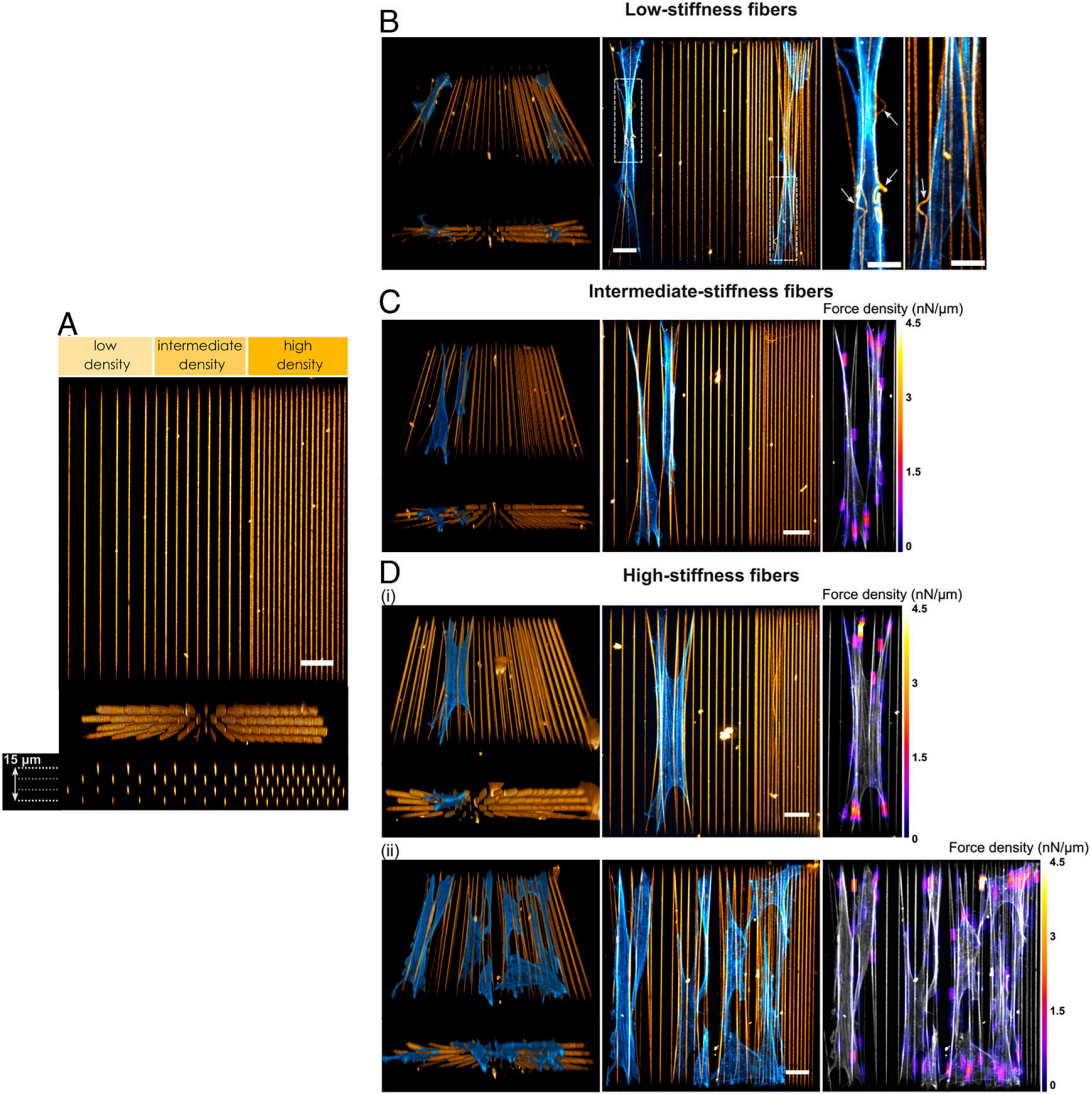
Application perspective: fiber density gradients. (*A*) Four-layer microscaffold incorporating a fiber density gradient. Fiber spacing is comprised between 5 μm and 15 μm within one layer, and successive layers are laterally shifted by half the fiber spacing (*Top*: maximum projection, Scale bar: 20 μm; *Center*: side view; *Bottom*: orthogonal plane; yellow: fibers). (*B*) NIH/3T3 fibroblasts spreading on the fiber density gradient, here on low-stiffness fibers (T_exp_ = 600 μs, L = 180 μm, k = 1.6 nN/μm) (*Center*: maximum projection, Scale bar: 20 μm; *Left*: 3D reconstruction and side view; *Right*: magnified view of the two regions outlined by a white dashed line, with arrows indicating fiber buckling, Scale bar: 10 μm; blue: actin, yellow: fibers). (*C*) NIH/3T3 fibroblasts in the low-density area, here on intermediate-stiffness fibers (T_exp_ = 700 μs, L = 180 μm, k = 3.8 nN/μm) (*Center*: maximum projection, Scale bar: 20 μm; *Left*: 3D reconstruction and side view; *Right*: maximum projection of the reconstructed force map; blue: actin, yellow: fibers). (*D*) NIH/3T3 fibroblasts on high-stiffness fibers (T_exp_ = 800 μs, L = 180 μm, k = 8.3 nN/μm) (*i*) Single cell spreading at the interface between low- and intermediate-density areas (*Center*: maximum projection, Scale bar: 20 μm; *Left*: 3D reconstruction and side view; *Right*: maximum projection of the reconstructed force map; blue: actin, yellow: fibers). (*ii*) Multiple cells spreading on the fiber density gradient microscaffold at higher cell density (center: maximum projection, Scale bar: 20 μm; *Left*: 3D reconstruction and side view; *Right*: maximum projection of the reconstructed force map; blue: actin, yellow: fibers).

To demonstrate the capability of our approach for measuring forces in more complex fiber architectures, we present an example of a fiber scaffold with a controlled density gradient. This scaffold consisted of four 180 μm-long fiber layers, organized into three regions with varying fiber densities. The interfiber lateral spacing decreased from 15 μm in the low-density region to 10 μm in the intermediate-density region and 5 μm in the high-density region. Each layer was vertically spaced by 5 μm, with successive layers laterally shifted to create a staggered arrangement (Fig. 5*A*). We seeded and fixed NIH/3T3 fibroblasts onto these scaffolds for several fiber stiffness conditions. Similar to the fiber arrays presented for macrophages, combining an increased fiber length with low exposure time (T_exp_ = 600 μs) allowed us to obtain very low stiffness fibers (Fig. 5*B*). Interestingly, we observed fiber buckling under these very low stiffness conditions, highlighting the contribution of axial cellular forces generated by fibroblasts. Since fiber buckling is not accounted for in our current mechanical model, our force reconstruction pipeline was not applied to this limiting case (Fig. 5*B*). In the case of intermediate (T_exp_ = 700 μs, Fig. 5*C*) and high exposure times (T_exp_ = 800 μs, Fig. 5 *D*, *i* and *ii*), we reconstructed the forces exerted in different regions of the fiber density gradient. We anticipate that these synthetic designs could be valuable for fundamental studies on directed cell migration in response to local density—topotaxis—and stiffness—durotaxis. Additionally, they may serve as models for applied biological research, such as investigating cell behavior at ECM density interfaces, which are relevant in physiological and pathological contexts, such as tumor boundaries.

## Discussion

In this study, we present a technique for fabricating multilayer arrays of fibers tailored for traction force measurements. The first contribution of our approach is the development of experimental procedures to obtain multilayer arrays of highly deformable fibers using two-photon polymerization (TPP). The second contribution lies in combining these fiber arrays with an integrated approach for measuring local 3D forces exerted by cells within the scaffolds, providing precise spatial localization of forces and enabling automation of the method. The precise control over 3D geometry and local chemistry afforded by this method enabled us to confine individual cells within simple, defined arrays of microfibers to measure their adhesion and force generation in a 3D fibrillar environment. We demonstrate that the stiffness of the fabricated fibers can be finely tuned across a broad range of values by adjusting polymerization parameters, making them well-suited for studying the deformations induced by various cell types. By developing image analysis tools along with mechanical modeling and a force inference pipeline, we generated 3D traction force maps at the scale of individual fibers, and we computed dynamic measurements of cell contractility. We validated our approach by applying it to two established models of adherent cells with high contractility, namely HUVECs (34–37) and NIH/3T3 cells (5, 29–33). Importantly, we observed how both fiber density and stiffness impact HUVEC adhesion and exerted forces in our 3D fiber system. In addition, our method could be used in conjunction with lattice light-sheet microscopy to measure weaker and highly dynamic forces exerted by immune cells such as macrophages and dendritic cells, and we showed that these latter amoeboid cells form transient anchors and short-lived protrusions that contact and deflect the fibers.

Compared to previous TPP-based systems, the low stiffness of the produced fibers greatly extends the range of measurable forces, with apparent fiber stiffness below 4 nN/µm achievable (Fig. 2*J*), enabling to capture the weak mechanical interactions characteristic of immune cells. To our knowledge, this study also presents the first fully integrated mechanical characterization by AFM of TPP-fabricated fiber elements, combining topographic scanning for detailed fiber profiling, nanoindentation for Young’s modulus measurements, and deflection of suspended fibers to assess apparent stiffness and shrinkage-induced tensile loading. Our method extends the force measurement capabilities of other existing microfabricated systems, such as micropillar arrays (18, 19) or suspended nanofibers platforms (20–22), which are typically limited to in-plane force detection, by enabling both in-plane and out-of-plane force measurements. Additionally, the use of laser stereolithography, compared to conventional soft lithography or fiber deposition techniques, significantly broadens the range of accessible 3D architectures and paves the way for studies in synthetic microenvironments that better capture the spatial complexity of native tissues. While the physiological realism of our system is reduced compared to 3D TFM, it offers a reductionist platform well-suited for deciphering elementary mechanisms of cell–fiber interactions within simplified systems with more controlled and homogeneous geometrical and mechanical properties. The reference-free nature of our platform also constitutes an advantage of our system compared to TFM, where plastic deformations occurring during experiments can compromise the recovered baseline configuration and significantly alter the analysis. Finally, unlike conventional TFM, which relies on bulk continuum mechanics, our platform enables the computation of cell traction forces at the scale of individual fibers. This makes it particularly well-suited for investigating cellular processes that involve fine-scale mechanical interactions with discrete fibers, for instance via protrusive structures such as filopodia or podosomes.

An important point to consider is how the dimensions, nanotexturation, and mechanical properties of the photopolymerized fibers compare to natural matrices. Collagen fibers in vivo, for example, have a wide range of diameters, ranging from several tenths of nanometers for single fibrils (59) to several microns for bundled fibers (60). In our set-up, chemical and polymerization parameters control the resolution, anisotropy, and nanostructuration of the fibers produced. Fibers reported in this work have adjustable dimensions of the same order as those of the extracellular matrix. However, we observed an asymmetry in the fiber cross-section, inherent to the anisotropic nature of the two-photon polymerization process. Although some works utilizing two-photon polymerization have reported features in the *z*-axis smaller than 500 nm (38, 61), ensuring stable ultrathin long fiber arrays at this scale remains a challenge that would require further design on resin chemistry, mechanics, and scaffold architecture. Furthermore, periodic nanostructuring similar to the D-band periodicity (62, 63) observed in physiological collagen I fibrils—typically around 67 nm—was seen in our fibers by AFM. In our case, the nanostructuration depends on the photopolymerization scanning speed and could likely be modulated to produce either smaller periods similar to those of collagen fibers, or very smooth fibers like those that are typically produced with bioengineering techniques like electrospinning. This flexibility could allow to further study the effect of local nanotexturation on cell response, with potential implications on curvature sensing (64), adhesion formation (65), and protrusion dynamics (66). Finally, AFM indentation of the photopolymerized fibers revealed Young’s moduli in the 1 to 40 MPa range, comparable to values reported for single hydrated collagen fibrils (67, 68). However, in both cases, the apparent mechanical response is not solely dictated by the intrinsic modulus. In our case, the apparent fiber stiffness is largely governed by the extensive axial stress generated during the development process, which can far exceed the material’s baseline modulus. Similarly, biological fiber networks often exhibit prestress in vivo, arising from the contractile activity of cells such as fibroblasts and myofibroblasts (69, 70). While this makes direct comparisons of the experienced stiffness difficult, the parallel also underscores how photopolymerized fibers can mimic key mechanical features of biological matrices, including the coupling between prestress and local stiffness.

A current limitation of our approach is that while it successfully captures lateral forces exerted by cells, which are crucial for matrix remodeling, it does not account for axial forces, i.e., forces that cells exert along the fiber axis. The axial stiffness of a beam under tensile stress is independent of the stored tension and depends solely on the fiber’s structural properties, given by $k_{\mathrm{axial}}=\frac{\mathrm{AE}}{L}$ where *A* is the fiber cross-section, *E* its Young’s modulus and *L* the fiber length. Based on our measurements of fiber properties, we estimate the axial stiffness to range between 16 nN/μm and 45 nN/μm for low- and high-stiffness 80 μm fibers, respectively. This is comparable to the range of lateral fiber stiffness that we measured, and it is therefore likely that substantial forces are also applied along the fiber axis. This is further supported by residual force analysis (*SI Appendix*, *Supplementary Note 3*). The inability to capture axial forces restricts the study of axial phenomena in our system, such as fiber buckling, as illustrated in Fig. 5*B*. While our method remains robust for quantifying forces from localized lateral protrusions, neglecting axial contributions leads to an underestimation of total cellular contractility. This limitation is particularly relevant in parallel fiber arrays and for large adherent cells, which tend to align along the fiber axis and may exert substantial axial forces. In contrast, orthogonal fiber arrangements tend to promote lateral interactions, as cells extend protrusions sideways. In such configurations, a more systematic computation of residual forces—especially when cells span two orthogonal fiber layers—could help mitigate the impact of this limitation. Furthermore, loosely adherent amoeboid cells, which display minimal axial elongation, may represent an interesting cell model for further investigation within our system. Quantifying the axial contribution could be achieved by embedding fluorescent nanoparticles within the fiber resin to track local deformations but this approach would require imaging the undeformed reference state, thereby eliminating a key advantage of our reference-free method. Alternatively, evolving toward more complex 3D scaffolds incorporating crosslinks between fibers, that could be tracked in every direction, appears as the most appealing prospect for future studies.

A fundamental advantage of our approach is its ability to modulate collective fiber organization in 3D. In the current study, we used two perpendicular layers of aligned fibers to create a simple scaffold for measuring 3D forces. Future developments involve engineering more intricate and physiologically relevant architectures, such as crosslinked fiber networks with diverse 3D fiber orientations. Compared to conventional reconstituted hydrogels, two-photon polymerization offers much finer control over key local network parameters such as fiber orientation, length, and connectivity. Expanding 3D force measurements to these more complex fiber architectures could therefore provide a valuable experimental platform for validating computational models of cell migration and force generation within discrete fibrous environments (71). This approach also offers opportunities for biological insights. Specifically, it may prove useful to mimic the complex fiber arrangements observed in peritumoral matrices, known as tumor-associated collagen signatures (TACS) (72), which feature spatial gradients in fiber density, stiffness, and orientation. We have shown that our technique allowed modulation of fiber stiffness (Fig. 2*J*), generation of fiber density gradients (Fig. 5), and recreation of distinct TACS-like architectures (e.g., aligned vs. disordered) to investigate how T lymphocytes respond to matrix topography (73). Additionally, the versatility of two-photon polymerization chemistry offers further development opportunities. While our current system uses purely elastic PEGDA fibers, our photopolymerization strategy could be expanded to incorporate viscoelasticity, which is central in morphogenesis or oncogenesis (74). This might be achieved by chemically adjusting crosslink strength or, alternatively, by photopolymerizing natural polymers such as ECM proteins, which exhibit viscoelastic behavior. The ability to combine 3D fiber architectures with diverse chemistries holds significant potential for creating synthetic 3D tumor microenvironment models to study the effect of local physical cues on cancer, immune, and stromal cells.

## Materials and Methods

### Photopolymerization Setup.

The two-photon polymerization set-up (Microlight3D, Grenoble, France) consisted of a QSwitch Teem Photonics laser (Grenoble, France), 10 kHz, 5 ns pulses, 10 µJ, 532 nm, a IX70 microscope with a water objective 60× (NA 1.2) LPlanApo, Olympus, and an oil objective 100× (NA 1.4), a piezo-*z* stage and a 3D stage (Physik Instrumente, Karlsruhe, Germany), and a Guppy CCD camera for monitoring structure formation. It was driven by Lithos software, with an autofocus module.

### Microscaffolds Preparation.

The cell-repellent Resin 1 was made of a mixture of PEGDA (Mn = 575 g/mol, Sigma Aldrich) with 15% (w/w) PETA (Sigma Aldrich) and 5% (w/v) 2,2-Dimethoxy-2-phenylacetophenone (Irgacure 651, Sigma Aldrich; see *SI Appendix*, Fig. S11). The cell-adhesive Resin 2 was made of a mixture of PEGDA (Mn = 250 g/mol, Sigma Aldrich) with 10% (w/w) PETA and 5% (w/v) Irgacure 651. We checked for selective cell adhesion using simple 2D composite structures (*SI Appendix*, Fig. S2).

Adhesion of acrylic-based photoresists to the glass substrate surface was enhanced by functionalizing the 30 mm glass coverslips with 3-(trimethoxysilyl)propyl methacrylate (Sigma Aldrich; 1 mM in ethanol) for 5 min. Walls of the microscaffold were fabricated with Resin 1 using a 60× water objective (NA 1.2) using a power of 6.4 mW. The excess Resin 1 was removed by washing the coverslip with pure ethanol and air-dried for 1 min. Resin 2 was then dropcast on top of the fabricated walls. Repositioning of the sample was handled by fabricating two landmarks with Resin 1 during the first step. Fibers were built using a 100× oil objective (NA 1.4) and a power of 3.9 mW. The coverslip was then placed in an incubation chamber and immersed in ethanol to remove the excess Resin 2 and sterilize the sample. This configuration allows the sample to be kept in liquid conditions at all times to prevent collapse of the fibers due to surface tension effects. The sample was then immersed in Phosphate Buffer Solution (PBS) by performing 10 successive partial washings of half the volume of the incubation chamber. The sample was then incubated in fibronectin (Sigma Aldrich, 10 µg/mL) coupled with CF™ 640R succinimidyl ester (Sigma Aldrich) at 37 °C for 1 h. Finally, the sample was immersed in cell medium by performing 10 successive partial washings.

### AFM Experiments.

All AFM experiments were performed using a commercial AFM (NanoScope VIII MultiMode AFM, Bruker Nano Inc- Nano Surfaces Division, Santa Barbara, CA). Glass substrates were fixed on a steel sample puck using double-sided tape. All experiments were performed at room temperature (~22 °C) in a PBS adjusted at pH 7.4. The cantilevers and samples were left to equilibrate for about 30 min in PBS before starting the measurements.

#### Imaging and nanoindentation.

Prior to imaging, long fibers (L = 300 μm) were photopolymerized near the glass substrate (3 μm above the coverslip) and collapsed during the washing steps. The sample was then immersed in PBS and all the subsequent steps were performed in PBS. AFM images were recorded using ozonated oxide-sharpened microfabricated Si_3_N_4_ AFM tip purchased from Bruker (SCANASYST-AIR, Nano Inc., Nano Surfaces Division, Santa Barbara, CA). For nanoindentation measurements, the spring constant of the cantilever was measured using the thermal noise method, yielding a value of 0.69 N/m. The curvature radius of silicon nitride tips was about 2 nm (manufacturer specifications). Series of measurements were performed with a constant indentation force of 1 nN. 16 × 16 windows were used with a scan size of 2 to 5 μm centered around the fiber. The Nanoscope Analysis software (Bruker Nano Inc- Nano Surfaces Division, Santa Barbara, CA) was used to automatically extract the contact point. Poisson ratio was set to 0.5 and the Sneddon model was used to determine the elastic modulus. The applied force was low enough to keep the indentation small and avoid probing the underlying glass substrate (*SI Appendix*, Fig. S6).

#### Fiber deflection.

For mechanical measurements on suspended fibers, a simplified version of the scaffold was designed with only three fibers, suspended 10 µm above the glass substrate. The samples were kept immersed in PBS at all times to avoid fibers collapsing due to surface tension effects. The mechanical measurements were performed using AFM tipless cantilevers, purchased from Bruker (NP-O10, Nano Inc., Nano Surfaces Division, Santa Barbara, CA). The spring constants of the cantilevers were measured using the thermal noise method, yielding values ranging between 0.13 N/m and 0.18 N/m. Series of measurements were performed using a 16 × 16 window without a lateral displacement of the cantilever, on the center of the suspended fibers, using a maximum force of 1 nN or 2 nN. The Nanoscope Analysis software (Buker Inc.) was used to automatically extract the contact point using a linear fit.

### Additional Materials and Methods.

Additional materials and methods regarding cell experiments, spinning disk and lattice light-sheet imaging, image analysis, and finite element modeling are available in *SI Appendix*, *SI Materials and Methods*.

## Supplementary Material

Appendix 01 (PDF)

Movie S1. Time lapse representing a NIH/3T3 Lifeact-GFP cell on a two-layer microscaffold (L = 80 μm) with highly deformable low-exposure fibers. The cell colormap codes for the z-position of the cell (purple = lower plane; blue = upper plane). Red: fibers. Total duration is 15 hours. Spinning-disk microscopy.

Movie S2. Time lapse representing a HUVEC Lifeact-GFP cell on a two-layer microscaffold (L = 80 μm) with highly deformable low-exposure fibers. The cell colormap codes for the z-position of the cell (purple = lower plane; blue = upper plane). Red: fibers. Total duration is 15 hours. Spinning-disk microscopy.

Movie S3. Time lapse representing HUVECs Lifeact-GFP on a two-layer large microscaffold (L = 200 μm) with highly deformable low-exposure fibers. The cell colormap codes for the z-position of the cell (purple = lower plane; blue = upper plane). Red: fibers. Total duration is 8 hours. Spinning-disk microscopy.

Movie S4. Time lapse representing HUVECs Lifeact-GFP on a two-layer large microscaffold (L = 200 μm) with moderately deformable high-exposure fibers. The cell colormap codes for the z-position of the cell (purple = lower plane; blue = upper plane). Red: fibers. Total duration is 8 hours. Spinning-disk microscopy.

Movie S5. Time lapse imaging of the development step following fiber polymerization during the microfabrication process. At t = 4 s, the monomer solution is washed with an excess of ethanol 100% and tension builds up within seconds. Total duration is 10 seconds.

Movie S6. 3D deflection map (left) and traction force map (right) of a NIH/3T3 cell on low-exposure fibers (T_exp_ = 600 μs, k = 3.5 nN/μm, L = 80 μm, lateral spacing = 10 μm). For both maps, a maximum projection, top view and side view are represented. Bottom: evolution of contractility over time. Total duration is 15 hours.

Movie S7. 3D deflection map (left) and traction force map (right) of a NIH/3T3 cell on medium-exposure fibers (T_exp_ = 700 μs, k = 8.3 nN/μm, L = 80 μm, lateral spacing = 10 μm). For both maps, a maximum projection, top view and side view are represented. Bottom: evolution of contractility over time. Total duration is 15 hours.

Movie S8. 3D deflection map (left) and traction force map (right) of a NIH/3T3 cell on high-exposure fibers (T_exp_ = 800 μs, k = 17.6nN/μm, L = 80 μm, lateral spacing = 10 μm). For both maps, a maximum projection, top view and side view are represented. Bottom: evolution of contractility over time. Total duration is 15 hours.

Movie S9. 3D deflection map (left) and traction force map (right) of a HUVEC cell on low-exposure fibers (T_exp_ = 600 μs, k = 3.5 nN/μm, L = 80 μm, lateral spacing = 10 μm). For both maps, a maximum projection, top view and side view are represented. Bottom: evolution of contractility over time. Total duration is 17 hours.

Movie S10. 3D deflection map (left) and traction force map (right) of a HUVEC cell on low-exposure fibers (T_exp_ = 600 μs, k = 3.5 nN/μm, L = 80 μm, lateral spacing = 5 μm). For both maps, a maximum projection, top view and side view are represented. Bottom: evolution of contractility over time. Total duration is 15 hours.

Movie S11. 3D deflection map (left) and traction force map (right) of a HUVEC cell on medium-exposure fibers (T_exp_ = 700 μs, k = 8.3 nN/μm, L = 80 μm, lateral spacing = 10 μm). For both maps, a maximum projection, top view and side view are represented. Bottom: evolution of contractility over time. Total duration is 9 hours.

Movie S12. 3D deflection map (left) and traction force map (right) of a HUVEC cell on high-exposure fibers (T_exp_ = 800 μs, k = 17.6 nN/μm, L = 80 μm, lateral spacing = 10 μm). For both maps, a maximum projection, top view and side view are represented. Bottom: evolution of contractility over time. Total duration is 17 hours.

Movie S13. 3D deflection map (left) and traction force map (right) of a macrophage on low-exposure fibers (T_exp_ = 600 μs, k = 1.8 nN/μm, L = 160 μm, lateral spacing = 5 μm). For both maps, a maximum projection, top view and side view are represented. Bottom: evolution of contractility over time. Total duration is 130 minutes.

Movie S14. 3D deflection map (left) and traction force map (right) of a macrophage on low-exposure fibers (T_exp_ = 600 μs, k = 1.8 nN/μm, L = 160 μm, lateral spacing = 10 μm). For both maps, a maximum projection, top view and side view are represented. Bottom: evolution of contractility over time. Total duration is 130 minutes.

Movie S15. 3D deflection map (left) and traction force map (right) of a macrophage on medium-exposure fibers (T_exp_ = 700 μs, k = 4.1 nN/μm, L = 160 μm, lateral spacing = 5 μm). For both maps, a maximum projection, top view and side view are represented. Bottom: evolution of contractility over time. Total duration is 130 minutes.

Movie S16. 3D deflection map (left) and traction force map (right) of a macrophage on high-exposure fibers (T_exp_ = 800 μs, k = 8.8 nN/μm, L = 160 μm, lateral spacing = 10 μm). For both maps, a maximum projection, top view and side view are represented. Bottom: evolution of contractility over time. Total duration is 130 minutes.

Movie S17. Time lapse representing a dendritic cell Lifeact-GFP cell on a two-layer microscaffold with low-exposure fibers (T_exp_ = 600 μs, k = 3.5 nN/μm, L = 80 μm, lateral spacing = 10 μm). Pink: F-actin, green: fibers. Total duration is 20 minutes. Lattice Light-Sheet microscopy.

Movie S18. 3D deflection map (left) and traction force map (right) of a dendritic cell on low-exposure fibers (T_exp_ = 600 μs, k = 3.5 nN/μm, L = 80 μm, lateral spacing = 10 μm), analysis of the Supplementary movie S17. For both maps, a maximum projection, top view and side view are represented. Bottom: evolution of contractility over time. Total duration is 20 minutes.

## Data Availability

The Matlab code developed for the analyses performed in this paper is available on GitHub: https://github.com/uclapierre/CellFLEX-FM (75). The data of this study are openly available in the following repository: https://doi.org/10.5281/zenodo.13646579 (76).
