# Supplementary material for: Quantifying cell traction forces at the single-fiber scale in 3D: An approach based on deformable photopolymerized fiber arrays
Source: Proc Natl Acad Sci U S A. 2025 Oct 13;122(42):e2507677122. doi: 10.1073/pnas.2507677122 (PMC12557527; doi:10.1073/pnas.2507677122)
Supplement: Supplementary file 1 — Appendix 01 (PDF) [file pnas.2507677122.sapp.pdf]

Supplementary Notes, Figures and Table

Table of contents

Supplementary Note 1 - Bending of a beam under extensive stress : analytical solution and FEM formulation 2

I. Bending of a beam under extensive stress 2

II. Computation of the stiffness matrix 4

III. Validation of the finite element model 9

Supplementary Note 2 - Evaluation of the force reconstruction optimization workflow and choice of regularization parameters 10

I. Unconstrained two-area loading without noise 11

II. Unconstrained two-area loading with noise - Regularization parameters 14

III. Constrained one-area loading with noise 16

Supplementary Note 3 - Residual forces allow to evaluate the underestimation of traction forces due to neglecting the axial component 19

Supplementary Note 4 – Experimental validation of the mechanical model 20

Supplementary Note 5 – MATLAB module for fiber deflection calculation 22

Supplementary Figures 24

Supplementary Table 30

Materials and Methods 35

List of Supplementary Movies 40

Supplementary Note 1 - Bending of a beam under extensive stress : analytical solution and FEM formulation

I. BENDING OF A BEAM UNDER EXTENSIVE STRESS

In this section, we study the response of a beam that is held under an extensive stress T at both ends to a point load P applied at a position $X = C$ along the beam longitudinal axis X, as represented on Sup.Fig.N1.1. To do so, we follow the steps that are detailed in paragraph 4 of Ref. [1]. The load induces a deformation of the beam $Y(X)$ perpendicular to the beam axis. At $X = 0$ (resp. $X = L$), the beam is subjected to an external moment M_0 (resp. M_1). The equation for the balance of the moments is

$$EI\frac{d^2Y}{dX^2} = TY - P\left(1 - \frac{C}{L}\right)X + M_0 \qquad \text{if } X < C, \tag{S1}$$

$$EI\frac{d^2Y}{dX^2} = TY - P\left(1 - \frac{X}{L}\right)C + M_1 \qquad \text{if } X > C, \tag{S2}$$

$$Y(0) = Y(L) = 0, \tag{S3}$$

$$\lim_{X\to C^-} Y(X) = \lim_{X\to C^+} Y(x), \tag{S4}$$

$$\lim_{X\to C^-} \frac{dY}{dX}(X) = \lim_{X\to C^+} \frac{dY}{dX}(X), \tag{S5}$$

where we introduced the Young modulus E and the moment of inertia I of the beam. In the following, we use the distance L between the ends of the beam as the length scale, and the extensive stress T exerted on both ends of the beam as the stress scale. We define the dimensionless variables $x = X/L$, $y = Y/L$. The problem has three dimensionless parameters: $\alpha = \frac{L}{\lambda}$, where $\lambda = \sqrt{EI/T}$ is a characteristic length, $\beta = \frac{P}{T}$, and $c = C/L$. Finally, we define the two integration constants $m_0 = \frac{M_0}{TL}$ and $m_1 = \frac{M_1}{TL}$, which correspond to dimensionless moments. The resulting set of equations is

$$\frac{1}{\alpha^2}\frac{d^2y}{dx^2} - y = -\beta\,(1-c)\,x + m_0 \qquad \text{if } x < c, \tag{S6}$$

$$\frac{1}{\alpha^2}\frac{d^2y}{dx^2} - y = -\beta\,(1-x)\,c + m_1 \qquad \text{if } x > c, \tag{S7}$$

$$y(0) = y(1) = 0, \tag{S8}$$

$$\lim_{x\to c^-} y(x) = \lim_{x\to c^+} y(x), \tag{S9}$$

$$\lim_{x\to c^-} \frac{dy}{dx}(x) = \lim_{x\to c^+} \frac{dy}{dx}(x). \tag{S10}$$

The sum of the homogeneous and particular solution of Eqs. (S6) and (S7) is

$$y(x) = \begin{cases} C_1\cosh(\alpha x) + C_2\sinh(\alpha x) + \beta\,(1-c)\,x - m_0 & \text{if } x < c \\ C_3\cosh(\alpha(1-x)) + C_4\sinh(\alpha(1-x)) + \beta\,(1-x)\,c - m_1 & \text{if } x > c, \end{cases}. \tag{S11}$$

where the constant C_i must be determined by the boundary conditions. From $y(0) = 0$ and $y(1) = 0$, we respectively find $C_1 = m_0$ and $C_3 = m_1$. The continuity of $y(x)$ and its derivative $\frac{dy}{dx}(x)$ at $x = c$

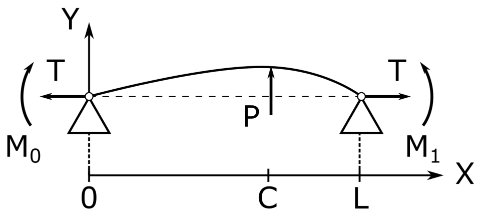

Sup.Fig.N1.1: Sketch of a beam subjected to a point load P applied at the position $X = C$. At its edges, the beam is subjected to an extensive stress T and a moment M_0 at $X = 0$ and M_1 at $X = L$.

allows to compute the constants C_2 and C_4. For simplicity, we use the linearity of the problem and write the solution as the sum of the solutions for β, m_0, and m_1 treated separately. Let us first consider the case $m_0 = m_1 = 0$. In this setting, the solution of the equation is

$$y_\beta(x) = \begin{cases} \beta\left[(1-c)\,x + C_{\beta,<}\sinh(\alpha x)\right] & \text{if } x < c \\ \beta\left[(1-x)\,c + C_{\beta,>}\sinh(\alpha(1-x))\right] & \text{if } x > c \end{cases}. \tag{S12}$$

with

$$C_{\beta,<} = -\frac{1}{\alpha(\cosh(\alpha c) + \coth(\alpha(1-c))\sinh(\alpha c))}, \tag{S13}$$

$$C_{\beta,>} = -\frac{1}{\alpha(\cosh(\alpha(1-c)) + \coth(\alpha c)\sinh(\alpha(1-c)))}. \tag{S14}$$

Let us now consider the case where $\beta = 0$ and $m_1 = 0$. In this setting, the solution of Eqs. (S6) to (S10) is

$$y_{m0}(x) = \begin{cases} m_0\left[\cosh(\alpha x) - 1 + C_{m0,<}\sinh(\alpha x)\right] & \text{if } x < c \\ m_0 C_{m0,>}\sinh(\alpha(1-x)) & \text{if } x > c \end{cases}. \tag{S15}$$

with

$$C_{m0,<} = \frac{\cosh(\alpha(1-c))}{\sinh(\alpha)} - \coth(\alpha), \tag{S16}$$

$$C_{m0,>} = \frac{1-\cosh(\alpha c)}{\sinh(\alpha)}. \tag{S17}$$

Conversely, the solution to the case $\beta = 0$ and $m_1 = 0$ is

$$y_{m1}(x) = \begin{cases} m_1 C_{m1,<}\sinh(\alpha x) & \text{if } x < c \\ m_1\left[\cosh(\alpha(1-x)) - 1 + C_{m1,>}\sinh(\alpha(1-x))\right] & \text{if } x > c \end{cases}. \tag{S18}$$

with

$$C_{m1,<} = \frac{1-\cosh(\alpha(1-c))}{\sinh(\alpha)}, \tag{S19}$$

$$C_{m1,>} = \frac{\cosh(\alpha c)}{\sinh(\alpha)} - \coth(\alpha). \tag{S20}$$

As the problem is linear, the full solution of Eqs. (S6) to (S10) is the sum $y(x) = y_\beta(x) + y_{m0}(x) + y_{m1}(x)$. From Eq. (S11), we have $C_2 = \beta C_{\beta,<} + m_0 C_{m_0,<} + m_1 C_{m_1,<}$ and $C_4 = \beta C_{\beta,>} + m_0 C_{m_0,>} + m_1 C_{m_1,>}$0.

In the following, we consider that the beam is clamped at both ends. Equivalently, the derivative of its displacement is null at these positions: $\frac{dy}{dx}(0) = \frac{dy}{dx}(1) = 0$. The two moments m_0 and m_1 are then determined by this additional constrain. By using Eq. (S11) and with explicit expressions for C_2 and C_4, we obtain the two following moments

$$m_0 = -\frac{\beta}{\alpha}\frac{(1-c+\alpha C_{\beta,>})C_{m1,>} - (c+\alpha C_{\beta,>})C_{m1,<}}{C_{m0,<}C_{m1,>} - C_{m0,>}C_{m1,<}}, \tag{S21}$$

$$m_1 = -\frac{\beta}{\alpha}\frac{(1-c+\alpha C_{\beta,>})C_{m0,>} - (c+\alpha C_{\beta,>})C_{m0,<}}{C_{m1,<}C_{m0,>} - C_{m1,>}C_{m0,<}}. \tag{S22}$$

When the load is applied at the center of the beam $c = 1/2$, these moments are equal by symmetry, and they can be expressed as $m_0 = m_1 = \frac{\beta \tanh(\alpha/4)}{2\alpha}$. The maximum is also located at the center of the beam and is

$$y_{\mathsf{max}} = \frac{\beta}{4}\left[1 - \frac{\tanh(\alpha/4)}{\alpha/4}\right]. \tag{S23}$$

In the limit where $\alpha/4 \gg 1$, we have $y_{\mathsf{max}} = \frac{\beta}{4}$ and $m_0 = m_1 = 0$, which corresponds to $Y_{\mathsf{max}} = \frac{PL}{4T}$. In the limit where $\alpha/4 \ll 1$, we have $y_{\mathsf{max}} = \frac{\beta\alpha^2}{192}$ with the non-zero moments $m_0 = m_1 = \frac{1}{2}\frac{\beta\alpha^2}{192}$, which corresponds to $Y_{\mathsf{max}} = \frac{PL^3}{192EI}$. The apparent stiffness is usually defined as $k_{\mathsf{app}} = \frac{P}{Y_{\mathsf{max}}}$. Using Eq.(S23) with dimensions yields

$$k_{\mathsf{app}} = \frac{4T/L}{1 - \frac{\tanh(L/4\lambda)}{L/4\lambda}}. \tag{S24}$$

In the two limits $\alpha/4 \ll 1$ and $\alpha/4 \gg 1$, we then respectively have $k_{\mathsf{app}} = \frac{4T}{L}$ and $k_{\mathsf{app}} = \frac{192EI}{L^3}$.

II. COMPUTATION OF THE STIFFNESS MATRIX

In this section, we compute the stiffness matrix associated with a beam under extensive stress using the formulation of Ref. [2]. The stiffness matrix $[K]$ relates the displacement and tangential angles associated to the forces and moments applied at any position along a beam. We use a finite element model where the beam is discretized into N nodes. Let us focus on the relation between two neighboring nodes that we call 1 and 2 separated by a distance $l = L/(N-1)$ and respectively located at $X = 0$ and $X = l$. For each node we denote U_i the displacement in the positive Y-direction, and θ_i the rotation angle of the beam along the Z-axis. We want to find the displacements U_1, U_2 and rotations θ_1, θ_2 caused by a given set of forces F_1, F_2 and moments M_1, M_2 applied at nodes 1, 2. Because we assume that the deflections are small, the rotation angle is given by the local derivative $\theta(x) = \frac{du}{dx}$. In the framework of Finite Element Model (FEM), the distance between nodes is assumed sufficiently small to have a linear relation between the forces and the moments and the displacements and the

rotations. Therefore,

$$\{F\} = \begin{pmatrix} F_1 \\ M_1 \\ F_2 \\ M_2 \end{pmatrix} = \begin{pmatrix} K_{11} & K_{12} & K_{13} & K_{14} \\ K_{21} & K_{22} & K_{23} & K_{24} \\ K_{31} & K_{32} & K_{33} & K_{34} \\ K_{41} & K_{42} & K_{43} & K_{44} \end{pmatrix} \begin{pmatrix} U_1 \\ \theta_1 \\ U_2 \\ \theta_2 \end{pmatrix} = [K]\{U\}, \tag{S25}$$

where we have introduced the stiffness matrix $[K]$. Note that the elements of this matrix do not have the same unit. The extension in the longitudinal direction of the beam and the rotation about the beam axis are not considered here.

The continuous beam deflection from node 1 to node 2 is denoted $U(X)$ and we must obey $U(0) = U_1$, $\frac{dU}{dX}(0) = \theta_1$, $U(l) = U_2$, and $\frac{dU}{dX}(l) = \theta_2$. The simplest function for $U(X)$ that is consistent with these boundary conditions is the cubic polynomial $U(X) = a_0 + a_1X + a_2X^2 + a_3X^3$. the coefficients a_i can be computed from the boundary conditions yielding

$$a_0 = U_1, \tag{S26}$$

$$a_1 = \theta_1, \tag{S27}$$

$$a_2 = -3\frac{U_1}{l^2} - 2\frac{\theta_1}{l} + 3\frac{U_2}{l^2} - \frac{\theta_2}{l}, \tag{S28}$$

$$a_3 = 2\frac{U_1}{l^3} + \frac{\theta_1}{l^2} - 2\frac{U_2}{l^3} + \frac{\theta_2}{l^2}. \tag{S29}$$

$$\tag{S30}$$

We can then write that the deformation is

$$U(X) = U_1 + \theta_1 X + \left(-3\frac{U_1}{l^2} - 2\frac{\theta_1}{l} + 3\frac{U_2}{l^2} - \frac{\theta_2}{l}\right) X^2 + \left(2\frac{U_1}{l^3} + \frac{\theta_1}{l^2} - 2\frac{U_2}{l^3} + \frac{\theta_2}{l^2}\right) X^3 \tag{S31}$$

$$= \underbrace{\left(1 - 3\frac{X^2}{l^2} + 2\frac{X^3}{l^3}\right)}_{N_1(X)} U_1 + \underbrace{\left(\frac{X}{l} - 2\frac{X^2}{l^2} + \frac{X^3}{l^3}\right) l}_{N_2(X)} \theta_1 + \underbrace{\left(3\frac{X^2}{l^2} - 2\frac{X^3}{l^3}\right)}_{N_3(X)} U_2 + \underbrace{\left(-\frac{X^2}{l^2} + \frac{X^3}{l^3}\right) l}_{N_4(X)} \theta_2 \tag{S32}$$

$$= \begin{bmatrix} N_1 & N_2 & N_3 & N_4 \end{bmatrix} \begin{bmatrix} U_1 \\ \theta_1 \\ U_2 \\ \theta_2 \end{bmatrix} \tag{S33}$$

$$= [N]\{U\}. \tag{S34}$$

Let us note that the X-dependency of U is now contained in $[N]$.

The Castigliano's theorem states that the force F_i (resp. the moment M_i) that is exerted on node i is the derivative of the total energy $\mathcal{E}_{\text{tot}}$ with respect to the displacement U_i (resp. the rotation θ_i) at node i. Here, we consider two contributions to the total energy. One is the strain energy $\mathcal{E}_b$ that is related to the bending rigidity of the beam, while the other is the energy $\mathcal{E}_s$ that is related to the extensive stress applied at both ends of the beam. From Castigliano's theorem, we have

$$\{F\} = \nabla\mathcal{E}_{\text{tot}} = \nabla(\mathcal{E}_b + \mathcal{E}_s) = ([K_b] + [K_s])\{U\} = [K]\{U\}, \tag{S35}$$

where we have introduced the operator $\nabla = (\frac{\partial}{\partial U_1}\frac{\partial}{\partial \theta_1}\frac{\partial}{\partial U_2}\frac{\partial}{\partial \theta_2})$ as well as the stiffness matrices $[K_b]$ and $[K_s]$, which will be defined later. Let us focus on the strain energy $\mathcal{E}_b$ first. The strain energy can be explicitly computed as (see Chap. 4 of Ref. [2])

$$\mathcal{E}_b = \frac{1}{2}\int dV E Y^2 \left(\frac{d^2U}{dX^2}\right)^2, \tag{S36}$$

$$= \frac{1}{2}E\underbrace{\left(\int Y^2 dA\right)}_{=I}\int_0^l \left(\frac{d^2[N]}{dX^2}\{U\}\right)^2 dX, \tag{S37}$$

$$= \frac{1}{2}EI\int_0^l \left(\frac{d^2N_1}{dX^2}U_1 + \frac{d^2N_2}{dX^2}\theta_1 + \frac{d^2N_3}{dX^2}U_2 + \frac{d^2N_4}{dX^2}\theta_2\right)^2 dX, \tag{S38}$$

where E is the Young modulus of the beam, and I is its moment of inertia. This gives

$$F_1 = \frac{\partial \mathcal{E}_b}{\partial U_1} = EI\int_0^l \left(\frac{d^2N_1}{dX^2}U_1 + \frac{d^2N_2}{dX^2}\theta_1 + \frac{d^2N_3}{dX^2}U_2 + \frac{d^2N_4}{dX^2}\theta_2\right)\frac{d^2N_1}{dX^2}dX. \tag{S39}$$

This expression can be related to Eq. (S25) and provides a way to compute the elements of the stiffness matrix K_{1i}. As an example, let us compute the element K_{11} appearing in Eq. (S25)

$$K_{11} = EI\int_0^l \left(\frac{d^2N_1}{dX^2}\right)^2 dX \tag{S40}$$

$$= \frac{EI}{l^3}\int_0^l \left(-6 + 12\frac{X}{l}\right)^2 \frac{dX}{l} \tag{S41}$$

$$= 12EI. \tag{S42}$$

Computing systematically the other elements K_{ii} of the bending stiffness matrix yields

$$[K_b] = \frac{EI}{l^3}\begin{pmatrix} 12 & 6l & -12 & 6l \\ 6l & 4l^2 & -6l & 2l^2 \\ -12 & -6l & 12 & -6l \\ 6l & 2l^2 & -6l & 4l^2 \end{pmatrix} \tag{S43}$$

Let us now turn to the potential energy $\mathcal{E}_s$ related to the extensive stress T applied at both ends of the beam. Using the expression found in Ref. [3], we have

$$\mathcal{E}_s = \frac{1}{2}\int_0^l T\left(\frac{dU}{dX}\right)^2 dX \tag{S44}$$

$$= \frac{1}{2}T\int_0^l \left(\frac{dN_1}{dX}U_1 + \frac{dN_2}{dX}\theta_1 + \frac{dN_3}{dX}U_2 + \frac{dN_4}{dX}\theta_2\right)^2 dX. \tag{S45}$$

To illustrate how to compute the stiffness matrix related to the extensive stress, we compute the moment M_1 applied on node 1. Using Castigliano's theorem

$$M_1 = T\int_0^l \left(\frac{dN_1}{dX}U_1 + \frac{dN_2}{dX}\theta_1 + \frac{dN_3}{dX}U_2 + \frac{dN_4}{dX}\theta_2\right)\frac{dN_2}{dX}dX. \tag{S46}$$

This expression can be related to Eq. (S25) and provides a way to compute the elements of the stiffness matrix K_{2i}. As an example, let us compute the element K_{21} appearing in Eq. (S25)

$$K_{21} = T\int_0^l \frac{dN_1}{dX}\frac{dN_2}{dX}dX \tag{S47}$$
$$= T\int_0^l \left(-6\frac{X}{l}+6\frac{X^2}{l^2}\right)\left(1-4\frac{X}{l}+3\frac{X^2}{l^2}\right)dx \tag{S48}$$
$$= \frac{T}{10} \tag{S49}$$

Computing the other elements K_{ii} of the bending stiffness matrix yields

$$[K_s] = \frac{T}{10l}\begin{pmatrix} 12 & l & -12 & l \\ l & \frac{4l^2}{3} & -l & -\frac{l^2}{3} \\ -12 & -l & 12 & -l \\ l & -\frac{l^2}{3} & -l & \frac{4l^2}{3} \end{pmatrix}. \tag{S50}$$

With the expressions of the stiffness matrices, we can rewrite Eq. (S35) in a dimensionalized form as

$$\{F\} = \begin{pmatrix} F_1 \\ M_1 \\ F_2 \\ M_2 \end{pmatrix} = \left(\frac{EI}{l^3}\begin{pmatrix} 12 & 6l & -12 & 6l \\ 6l & 4l^2 & -6l & 2l^2 \\ -12 & -6l & 12 & -6l \\ 6l & 2l^2 & -6l & 4l^2 \end{pmatrix} + \frac{T}{10l}\begin{pmatrix} 12 & l & -12 & l \\ l & \frac{4l^2}{3} & -l & -\frac{l^2}{3} \\ -12 & -l & 12 & -l \\ l & -\frac{l^2}{3} & -l & \frac{4l^2}{3} \end{pmatrix}\right)\begin{pmatrix} U_1 \\ \theta_1 \\ U_2 \\ \theta_2 \end{pmatrix} \tag{S51}$$

Let us extend this analysis to both Y and Z displacements of the beam. We introduce the displacement U_i^z in the Z-direction and its associated rotational angle θ_i^y about the Y-axis for each node i. Conversely, the displacement in the Y-direction is called U_i^y and its associated rotational angle about the Z-axis θ_i^z. The name of the forces and moments applied at each node i are changed accordingly. We define the stiffness matrices then obey

$$\{F\} = \begin{pmatrix} F_1^y \\ M_1^z \\ F_2^y \\ M_2^z \\ F_1^z \\ M_1^y \\ F_2^z \\ M_2^y \end{pmatrix} = \left([K_b^{2d}] + [K_s^{2d}]\right)\begin{pmatrix} U_1^y \\ \theta_1^z \\ U_2^y \\ \theta_2^z \\ U_1^z \\ \theta_1^y \\ U_2^z \\ \theta_2^y \end{pmatrix} = ([K_b^{2d}] + [K_s^{2d}])\{U\} \tag{S52}$$

We assume that motions in (U_i^y, θ_i^z) are decoupled from motions in (U_i^z, θ_i^y). Therefore,

$$[K_i^{2d}] = \begin{pmatrix} [K_i^{xy}] & [0] \\ [0] & [K_i^{xz}] \end{pmatrix}, \tag{S53}$$

where $i = b, s$, and $[0]$ is a (4×4) matrix with all components set to zero. Above, we computed the stiffness matrices for motions in the (X, Y)-plane, which holds true here: $[K_b^{xy}] = [K_b]$ and $[K_s^{xy}] = [K_s]$, with $[K_b]$ and $[K_s]$ respectively defined in Eqs. (S43) and (S50). To compute the

stiffness matrices $[K_b^{xz}]$ and $[K_s^{xz}]$ for motions in the (X,Z)-plane, we must adapt the above reasoning to our sign convention for the rotation angles. All the angles are defined positive for anticlockwise rotation about their reference axis, e.g. θ_1^z is positive for anti-clockwise rotation of the beam about the Z-axis at node 1. With right-handed convention for the (X,Y,Z) reference frame, if the longitudinal direction of the beam is along the positive X-axis, we have $\theta^z = \frac{dU^y}{dX}$, and $\theta^y = -\frac{dU^z}{dX}$. Therefore, the reasoning detailed above for (U_i^y, θ_i^z) must be changed for the motions in (U_i^z, θ_i^y) by using the transformation $(U_i^z, \theta_i^y) \to (U_i^z, -\theta_i^y)$ and $(F_i^z, M_i^y) \to (F_i^z, -M_i^y)$. With this transformation, we find that the stiffness matrix related to the bending rigidity is

$$[K_b^{2d}] = \frac{EI}{l^3}\begin{pmatrix} 12 & 6l & -12 & 6l & 0 & 0 & 0 & 0 \\ 6l & 4l^2 & -6l & 2l^2 & 0 & 0 & 0 & 0 \\ -12 & -6l & 12 & -6l & 0 & 0 & 0 & 0 \\ 6l & 2l^2 & -6l & 4l^2 & 0 & 0 & 0 & 0 \\ 0 & 0 & 0 & 0 & 12 & -6l & -12 & -6l \\ 0 & 0 & 0 & 0 & -6l & 4l^2 & 6l & 2l^2 \\ 0 & 0 & 0 & 0 & -12 & 6l & 12 & 6l \\ 0 & 0 & 0 & 0 & -6l & 2l^2 & 6l & 4l^2 \end{pmatrix}. \tag{S54}$$

By convention, the vectors $\{F\}$ and $\{U\}$ are usually shown in the following order $\{F\} = (F_1^y, F_1^z, M_1^y, M_1^z, F_2^y, F_2^z, M_2^y, M_2^z)^T$ and $\{U\} = (U_1^y, U_1^z, \theta_1^y, \theta_1^z, U_2^y, U_2^z, \theta_2^y, \theta_2^z)^T$. With , $[K_b^{2d}]$ becomes

$$[K_b^{2d}] = \frac{EI}{l^3}\begin{pmatrix} 12 & 0 & 0 & 6l & -12 & 0 & 0 & 6l \\ 0 & 12 & -6l & 0 & 0 & -12 & -6l & 0 \\ 0 & -6l & 4l^2 & 0 & 0 & 6l & 2l^2 & 0 \\ 6l & 0 & 0 & 4l^2 & -6l & 0 & 0 & 2l^2 \\ -12 & 0 & 0 & -6l & 12 & 0 & 0 & -6l \\ 0 & -12 & 6l & 0 & 0 & 12 & 6l & 0 \\ 0 & -6l & 2l^2 & 0 & 0 & 6l & 4l^2 & 0 \\ 6l & 0 & 0 & 2l^2 & -6l & 0 & 0 & 4l^2 \end{pmatrix}. \tag{S55}$$

Applying the same transformation to the stiffness matrix $[K_s^{2d}]$ that is related to the stress applied at both ends of the beam yields

$$[K_s^{2d}] = \frac{P}{10l}\begin{pmatrix} 12 & 0 & 0 & l & -12 & 0 & 0 & l \\ 0 & 12 & -l & 0 & 0 & -12 & -l & 0 \\ 0 & -l & \frac{4l^2}{3} & 0 & 0 & l & -\frac{l^2}{3} & 0 \\ l & 0 & 0 & \frac{4l^2}{3} & -l & 0 & 0 & -\frac{l^2}{3} \\ -12 & 0 & 0 & -l & 12 & 0 & 0 & -l \\ 0 & -12 & l & 0 & 0 & 12 & l & 0 \\ 0 & -l & -\frac{l^2}{3} & 0 & 0 & l & \frac{4l^2}{3} & 0 \\ l & 0 & 0 & -\frac{l^2}{3} & -l & 0 & 0 & \frac{4l^2}{3} \end{pmatrix}. \tag{S56}$$

We have calculated the matrices that describe the displacement of two neighboring nodes along the beam in response to forces and moments applied at each of those two nodes. These matrices can be extended to any pair of neighboring nodes along the beam. This provides a matrix that is diagonal by

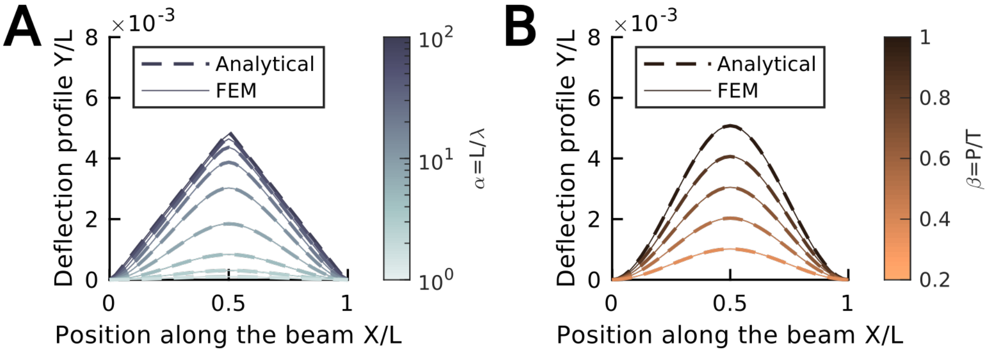

Sup.Fig.N1.2: Comparison of the displacement of a clamped beam in response to a point load P applied at $C = L/2$ obtained with the analytical (Section I, dashed line) and FEM (Section II, continuous line) calculation. The deflection profiles Y/L are plotted as a function of the normalized position X/L along the beam for varying adimensional parameters: **A**: $\beta = 0.02$, and α is varied logarithmically from 1 to 100 with a multiplicative factor of $10^{1/4}$, **B**: β is varied linearly from 0.2 to 1 with an increment of 0.2 and $\alpha = 1$. The beam was discretized into 41 nodes for all FEM calculations.

block, and that can describe the full beam if appropriate boundary conditions are provided at the beam ends.

III. VALIDATION OF THE FINITE ELEMENT MODEL

To validate the Finite Element Model (FEM) developed in Section II, we confront it to the analytical solution found in Section I for the case of a point load P applied at the center of the beam ($C = L/2$). Both ends of the beam are assumed to be clamped in the FEM model, matching the boundary conditions presented in Section I. The problem then depends only on the two adimensional parameters α and β defined in Section I (the third one c corresponds to the point where the load is applied and is here fixed to $c = 1/2$). To ensure a fast computational time (< 10 ms per run) and a small relative error ($< 3\%$) of the FEM calculation with respect to the analytical calculation, we choose to discretize the beam into 41 nodes. To describe the full beam using FEM, we adapted the package calfem (https://github.com/CALFEM/calfem-matlab) by changing the stiffness matrix of the beam3e function to the sum of the stiffness matrices in Eqs. (S55) and (S56). Sup.Fig.N1.2 shows that the FEM calculation matches the analytical one for any value of α (representative values shown on Sup.Fig.N1.2A, and any value of β (representative values shown on Sup.Fig.N1.2B).

[1] S. Timoshenko, *Strength of Materials. 2: Advanced Theory and Problems* (Van Nostrand, 1956).
[2] D. Hutton, *Fundamentals of finite element analysis* (McGraw-Hill, 2004).
[3] B. Tu-Sekine, A. Padhi, S. Jin, S. Kalyan, K. Singh, M. Apperson, R. Kapania, S. C. Hur, A. Nain, and S. F. Kim, The FASEB Journal **33**, 14137 (2019).

Supplementary Note 2 - Evaluation of the force reconstruction optimization workflow and choice of regularization parameters

I. Unconstrained two-area loading without noise

A common situation observed in our setup involved an elongated cell fully spread across several fibers, exerting two poles of traction forces at each end of the contacted fibers. Traction force recovery in this situation is more challenging than in the case of a localized loading exerted by a local protrusion, where we constrain the traction localization to a limited number of nodes at the intersection between the cell body and the fiber. Therefore, we chose this limiting case to optimize regularization parameters and assess the performance of our force reconstruction process.

In our simulation, an 80 µm-long fiber was divided into 41 nodes, and a 20 nN load was uniformly distributed along the *y* and *z* axes across two regions of 3 nodes each at *L*/6 and 5*L*/6 (Sup.Fig.N2.1A). We generated the corresponding deflection profiles and we used them to recover the applied forces using our optimization scheme.

1. Choice of *Ɛ*

The first parameter to set is *Ɛ,* the step size of the cost function gradient used in the optimization process:

$$\frac{\partial \mathrm{C}(\mathrm{p}_0,\dots,\mathrm{p}_\mathrm{i},\dots,\mathrm{p}_{\mathrm{Nb\_nodes}})}{\partial \mathrm{p}_\mathrm{i}} = \frac{\mathrm{C}\big(\mathrm{p}_0,\dots,\mathrm{p}_\mathrm{i}+\mathcal{E},\dots,\mathrm{p}_{\mathrm{Nb\_nodes}}\big) - \mathrm{C}\big(\mathrm{p}_0,\dots,\mathrm{p}_\mathrm{i},\dots,\mathrm{p}_{\mathrm{Nb\_nodes}}\big)}{\mathcal{E}}$$

where C is the optimization cost function and p is the load vector.
Larger values of *Ɛ* fail to converge to the expected solution while smaller values lead to larger computation time. Sup.Fig.N2.2A, B illustrate the force profiles obtained for different values of *Ɛ*.

We define two error metrics (Sup.Fig. N2.1B, C):

- **Contractility error**, accounts for the deviation in total absolute value of the exerted forces between the simulation and the reconstruction:

$$\boldsymbol{Err_c} = \frac{\left|\sum_{k=1}^{Nb\_nodes}\left(|p^{true}{}_{k,y}| + |p^{true}{}_{k,z}|\right) - \sum_{k=1}^{Nb\_nodes}\left(|p^{reconstructed}{}_{k,y}| + |p^{reconstructed}{}_{k,z}|\right)\right|}{\sum_{k=1}^{Nb\_nodes}\left(|p^{true}{}_{k,y}| + |p^{true}{}_{k,z}|\right)}$$

- **Force profile error,** defined as the sum of the absolute deviation in force at each node, which penalizes both incorrect force intensity and incorrect force localization:

$$\boldsymbol{Err_{fp}} = \frac{\sum_{k=1}^{Nb\_nodes} (|p^{true}{}_{k,y} - p^{reconstructed}{}_{k,y}| + |p^{true}{}_{k,z} - p^{reconstructed}{}_{k,z}|)}{\sum_{k=1}^{Nb\_nodes} (|p^{true}{}_{k,y}| + |p^{true}{}_{k,z}|)}$$

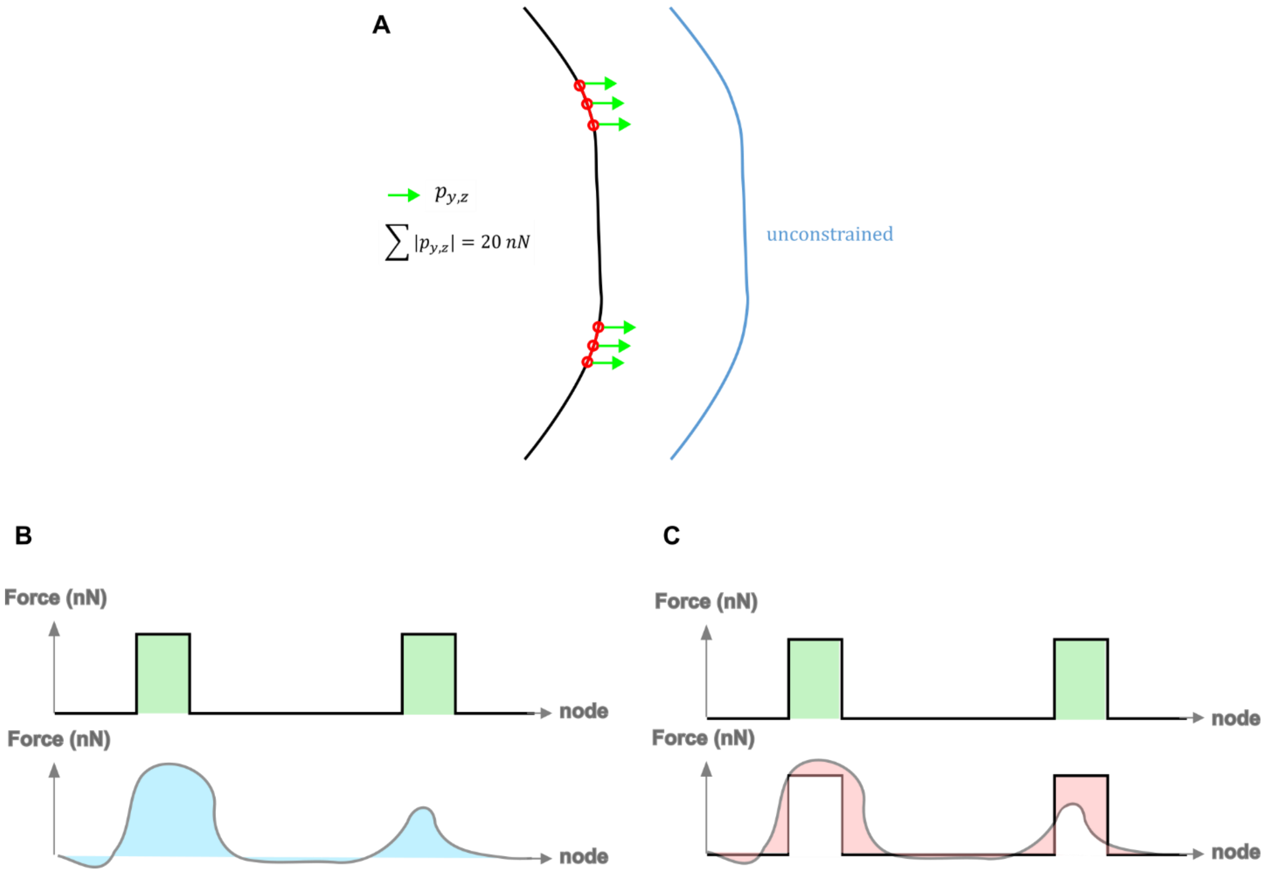

Supplementary Figure N2.1 - Simulation and error metrics

A. Simulated unconstrained two-area loading. **B.** The contractility error metric compares the simulated contractility (top, green area) and the reconstructed one (bottom, blue). **C.** The force profile error metric evaluates the local underestimation or overestimation of the reconstructed force (bottom, red area).

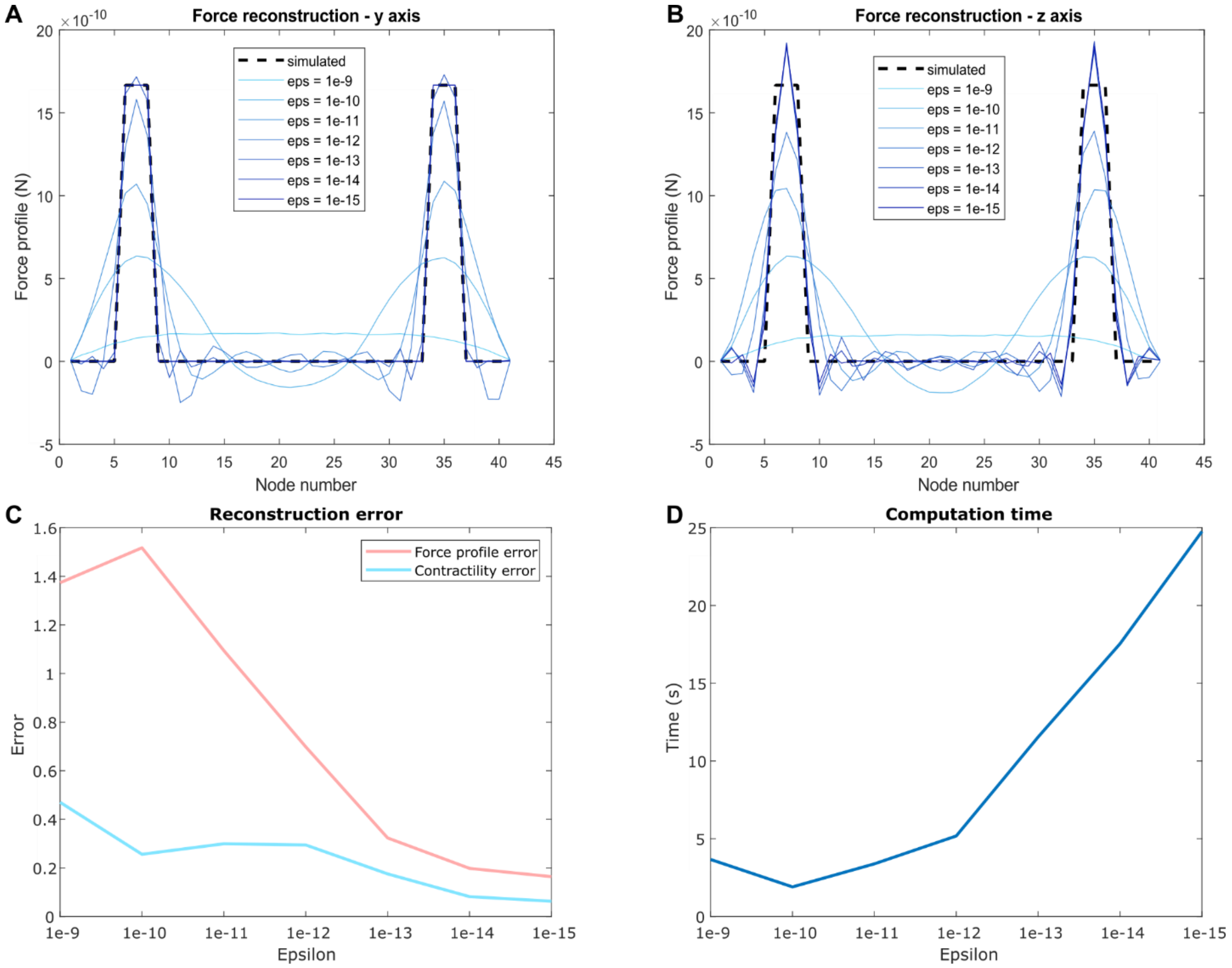

Supplementary Figure N2.2 – Optimization of the gradient step size $\mathcal{E}$

A. Simulated force profile and corresponding y component and **B.** z component of the reconstructions for different values of the gradient step size $\mathcal{E}$. **C.** Reconstruction errors and **D.** computation time for the force recovery process for different $\mathcal{E}$ values.

Based on the observation of these reconstruction errors and of the computation time, we chose to set Ɛ = 10^{-13} N.

2. Influence of the number of nodes on the reconstruction

In this section, we aim to evaluate how the number of nodes chosen in the Finite Element discretization of fibers affects the performance of the traction force recovery. We simulated a loading pattern similar to the one described in section 1, where the loading area had a fixed length of 6 µm. We generated the corresponding "ideal" deflection profile using a large number of nodes (Nb_nodes = 201). This deflection profile was then subsampled to a lower number of nodes, and force reconstruction was performed using Ɛ = 10^{-13} N. The number of nodes was varied between 5 and 77 (Sup.Fig.N2.3A, B).

For each case, we computed the contractility error and force profile error (Sup.Fig.N2.3C) as well as the computation time (Sup.Fig.N2.3D). We confirmed our choice of Nb_nodes = 41 as a good compromise between reconstruction accuracy and computation time, achieving an excellent agreement between the simulated and reconstructed deflection profiles (Sup.Fig.N2.3E, F). It is worth noting that due to the cross-section anisotropy of the fiber, the moment of inertia differ along the *y* and *z* axes. Although the contribution of bending stiffness to the overall deflection is minor compared to tension, this leads to slightly different deflection profiles along the *y* and *z* directions.

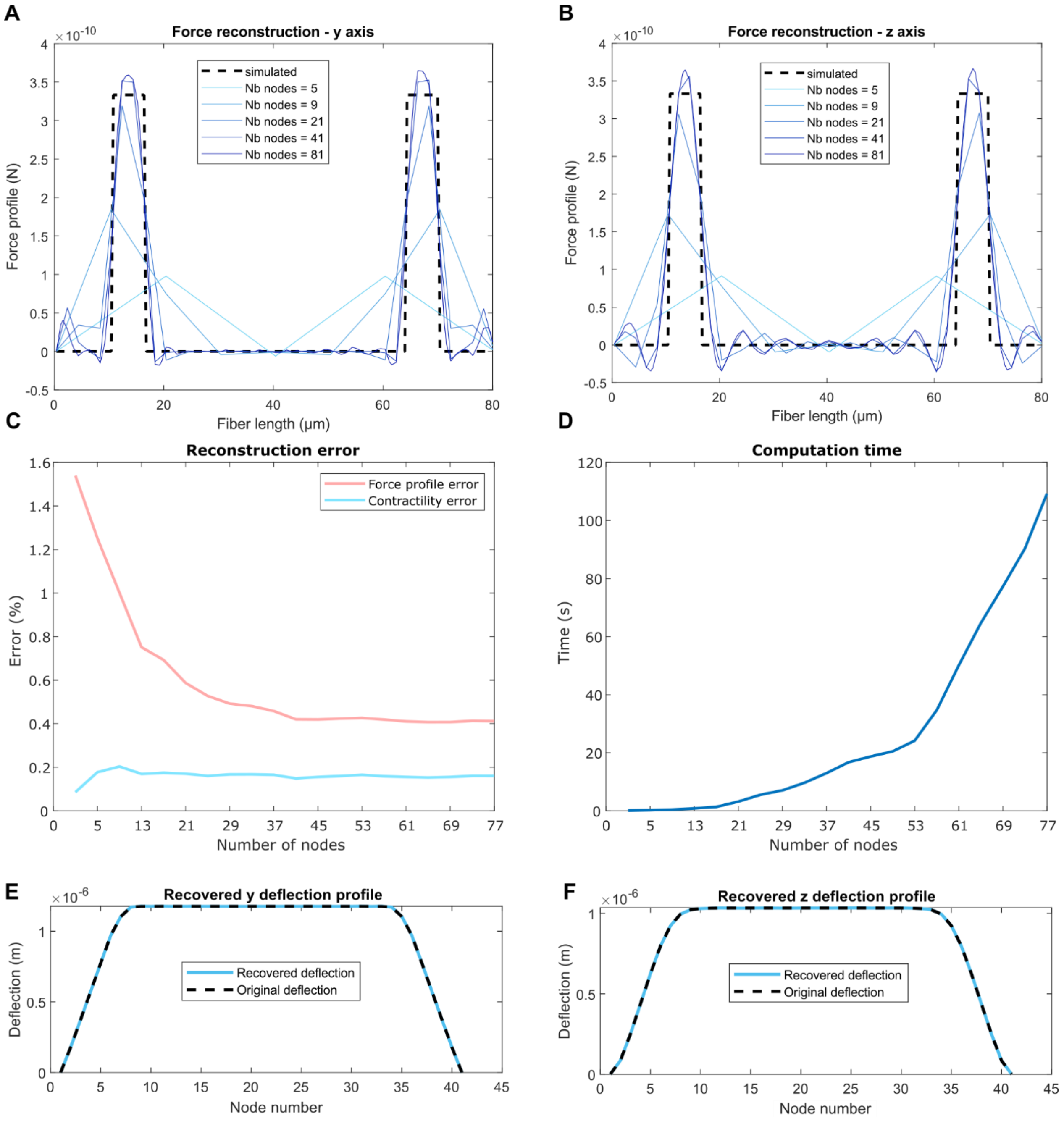

Supplementary Figure N2.3 – Influence of the number of nodes on the reconstruction

A. Simulated force profile and corresponding y component and **B.** z component of the reconstructions for different number of nodes. **C.** Reconstruction errors and **D.** computation time for the force recovery process for different number of nodes. **E.** Deflection profile corresponding to a reconstruction with $\mathcal{E}$ = 10^{-13} N and 41 nodes along the y direction and **F.** along the z direction.

II. Unconstrained two-area loading with noise - Regularization parameters

Unlike the ideal simulated case previously described, the deflection measurements extracted from our system exhibit some noise. The typical standard deviation is 0.3 µm, which corresponds to our final voxel size. To simulate the effect of this noise, we added a Gaussian noise (σ = 0.3 µm) to the simulated deflection profile before applying the inverse method for traction recovery. In this scenario, the recovered deflection profile clearly shows overfitting to the added noise (Sup.Fig.N2.4C, D) resulting in poor force reconstruction (Sup.Fig.N2.4A,B).

To adress this, we applied an elastic net regularization (L1+L2) to prevent overfitting. To select optimal regularization parameters λ_1 and λ_2, we ran the simulation multiple times (N=10) with various parameter pairs and calculated the average reconstruction errors (Sup.Fig.N2.4E, F). We identified the optimal pair ($\lambda_1=10^2$ m.N^{-1}, $\lambda_2=10^9$ m.N^{-2}) which minimized both errors, and used these parameters for all regularized reconstructions.

Examples of reconstructed force profiles with regularization are shown in Sup.Fig. N2.4G, H, along with the corresponding deflection profiles (Sup.Fig. N2.4I, J). While the reconstruction is significantly improved, these examples examples also illustrate some limitations encountered in this challenging case, including underestimation or overestimation of local force intensity and shifting of the force peak localization.

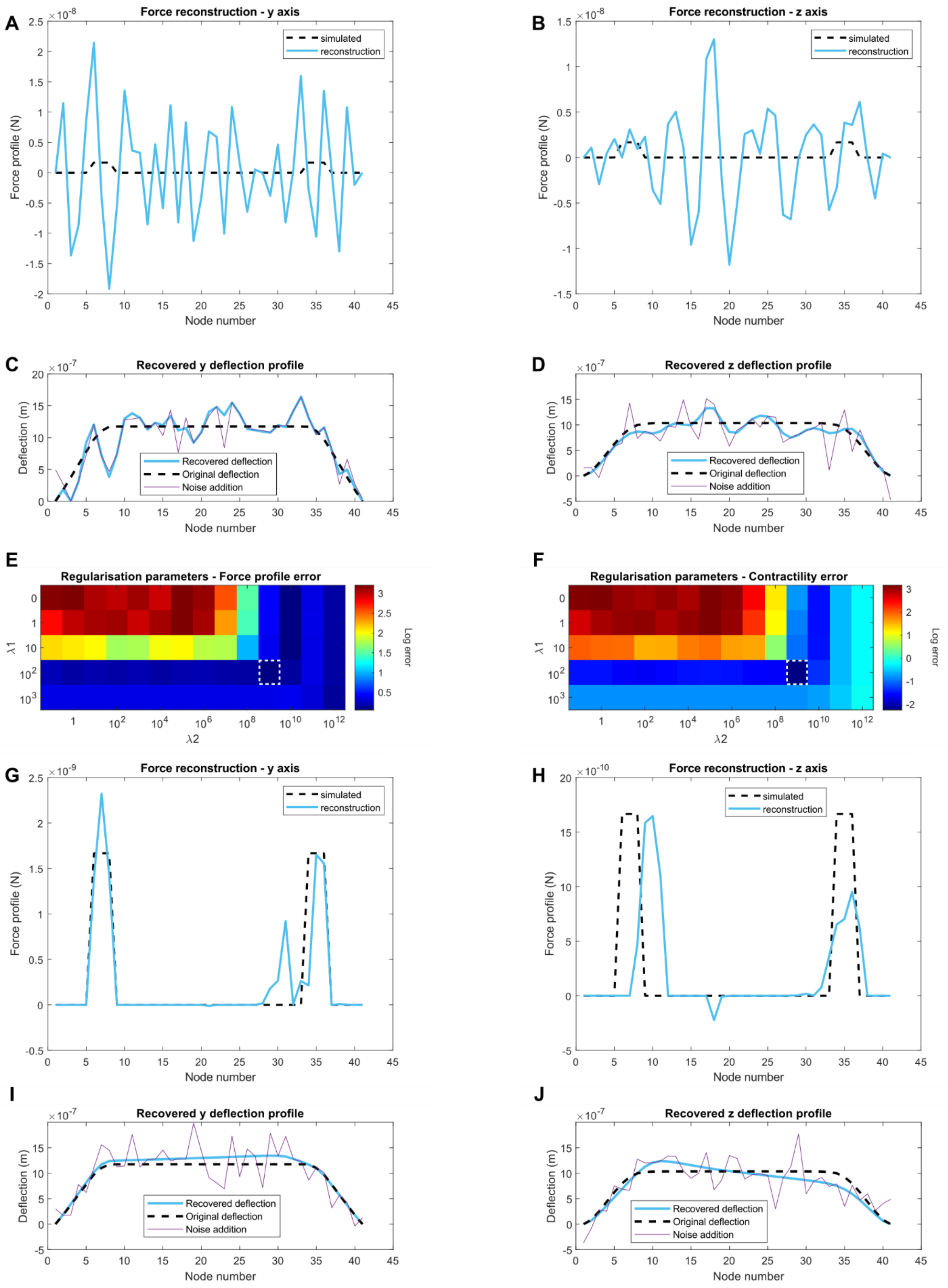
A
Force reconstruction - y axis
Force profile (N)
Node number
simulated
reconstruction
B
Force reconstruction - z axis
Force profile (N)
Node number
simulated
reconstruction
C
Recovered y deflection profile
Deflection (m)
Node number
Recovered deflection
Original deflection
Noise addition
D
Recovered z deflection profile
Deflection (m)
Node number
Recovered deflection
Original deflection
Noise addition
E
Regularisation parameters - Force profile error
λ1
λ2
Log error
F
Regularisation parameters - Contractility error
λ1
λ2
Log error
G
Force reconstruction - y axis
Force profile (N)
Node number
simulated
reconstruction
H
Force reconstruction - z axis
Force profile (N)
Node number
simulated
reconstruction
I
Recovered y deflection profile
Deflection (m)
Node number
Recovered deflection
Original deflection
Noise addition
J
Recovered z deflection profile
Deflection (m)
Node number
Recovered deflection
Original deflection
Noise addition

Supplementary Figure N2.4 - Unconstrained two-area loading with noise

A. Simulated force profile and corresponding y component and **B.** z component of the reconstructions with addition of a gaussian noise (σ = 0.3 µm) to the simulated deflection profile before the traction recovery process. **C.** Corresponding reconstructed deflection profiles along the y direction and **D.** along the z direction. **E.** Logarithmic representation of the force profile error and **F.** of the contractility error depending on the regularization parameters λ_1 and λ_2. The white dashed line square figures the pair of parameters that minimizes both the two reconstruction errors. **G.** Y component of the reconstructed force profile and **H.** z component after regularization. **I.** Corresponding reconstructed deflection profile along the y direction and **J.** along the z direction.

III. Constrained one-area loading with noise

To assess the performance of our force reconstruction framework in the case of a localized loading – such as an individual protrusion pulling on a fiber - we simulated a 20 nN loading distributed along the *y* and *z* axes and applied at a single node, located at *L*/4. A Gaussian noise (*σ* = 0.3 µm) was added to the simulated deflection profile. The regularized reconstruction of both the force and deflection profiles are shown in Sup.Fig. N2.5B, C and D,E, demonstrating excellent agreement between the simulation and the reconstruction in this simpler scenario.

We then evaluated the impact of the loading area on reconstruction performance by distributing the 20 nN load over an increasingly larger area, centered around *L*/4. The corresponding reconstruction errors and computation times are displayed in Sup.Fig.N2.5F, G.

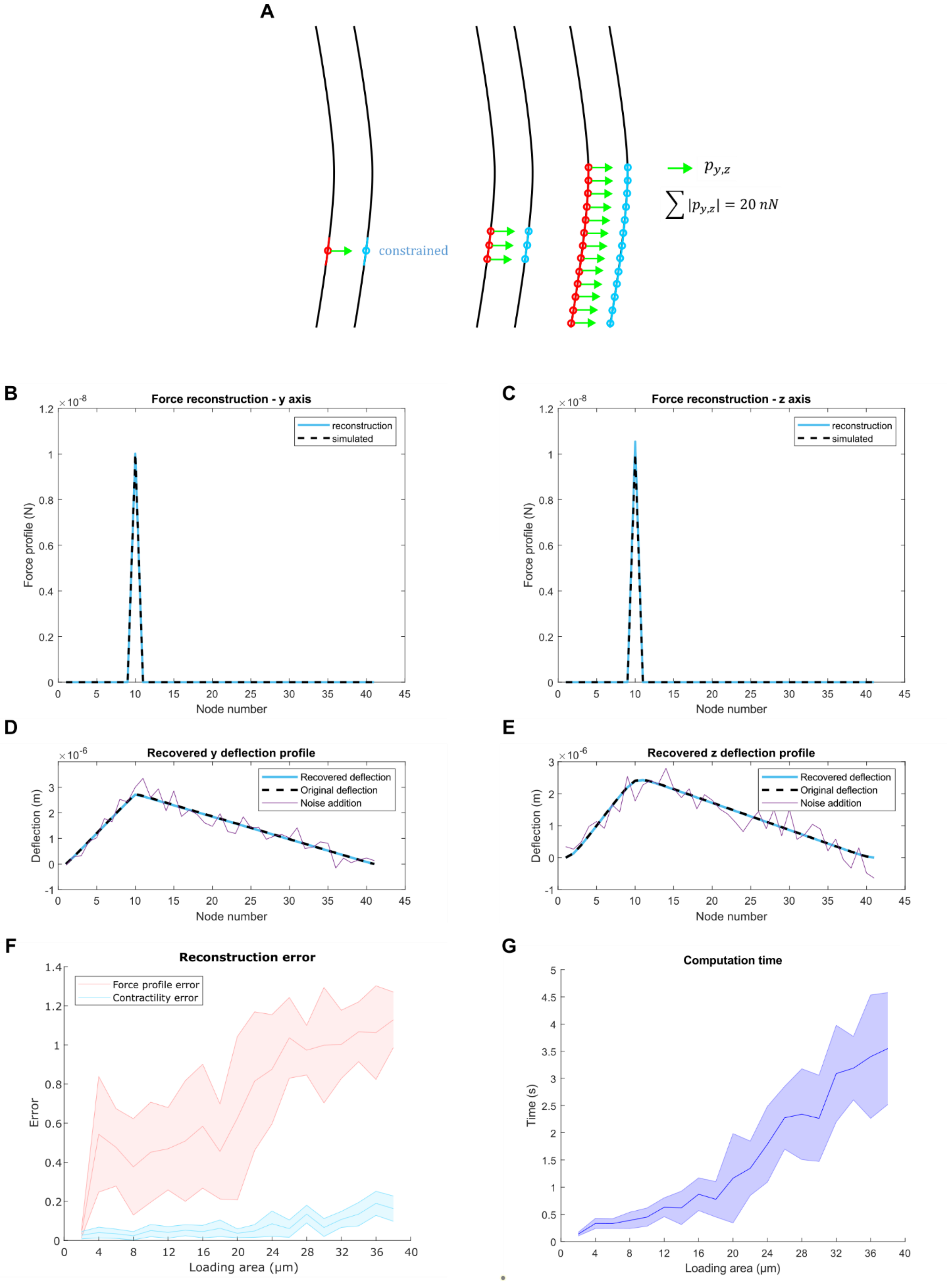
A
constrained
$p_{y,z}$
$\sum |p_{y,z}| = 20\ nN$
B
Force reconstruction - y axis
reconstruction
simulated
Force profile (N)
Node number
C
Force reconstruction - z axis
reconstruction
simulated
Force profile (N)
Node number
D
Recovered y deflection profile
Recovered deflection
Original deflection
Noise addition
Deflection (m)
Node number
E
Recovered z deflection profile
Recovered deflection
Original deflection
Noise addition
Deflection (m)
Node number
F
Reconstruction error
Force profile error
Contractility error
Error
Loading area (μm)
G
Computation time
Time (s)
Loading area (μm)

Supplementary Figure N2.5 - Constrained one-area loading with noise

A. Simulated constrained one-area loading distributed over an increasing number of nodes. **B.** Simulated force profile and corresponding y component and **C.** z component of the reconstructions for a localized loading applied at L/4. **D.** Corresponding reconstructed deflection profiles along the y direction and **E.** along the z direction. **F.** Reconstruction errors and **G.** computation time of the traction recovery process as a function of the loading area (1 node = 2 µm) for a constant total contractility of 20 nN.

Supplementary Note 3 - Residual forces allow to evaluate the underestimation of traction forces due to neglecting the axial component

Cell traction forces should sum to zero[1]. We quantified the residual forces, defined as the absolute value of the summed x-y components $|\sum p_{x,y}|$ or z component $|\sum p_z|$ of the traction forces at a given time point. We observed that when a cell contacts fibers on the two orthogonal planes, the x-y component of the residual forces often peaks, indicating an apparent force imbalance due to neglecting the axial component. In this case (Sup.Fig.N3.1), the bending of fibers in one plane is counterbalanced by an axial force exerted on the orthogonal layer. Analyzing the residual force components allows us to estimate the contribution of axial forces in certain cases. For example, in the scenario shown in Sup.Fig.N3.1 (T_1) , this contribution reached up to 65% of the measured contractility during contractile bursts. During phases where the cell contacted only one fiber layer (T_2), the x-y component of the residual forces remained close to zero. It is important to note that this approach does not account for axial forces balanced within each independent fiber layer.

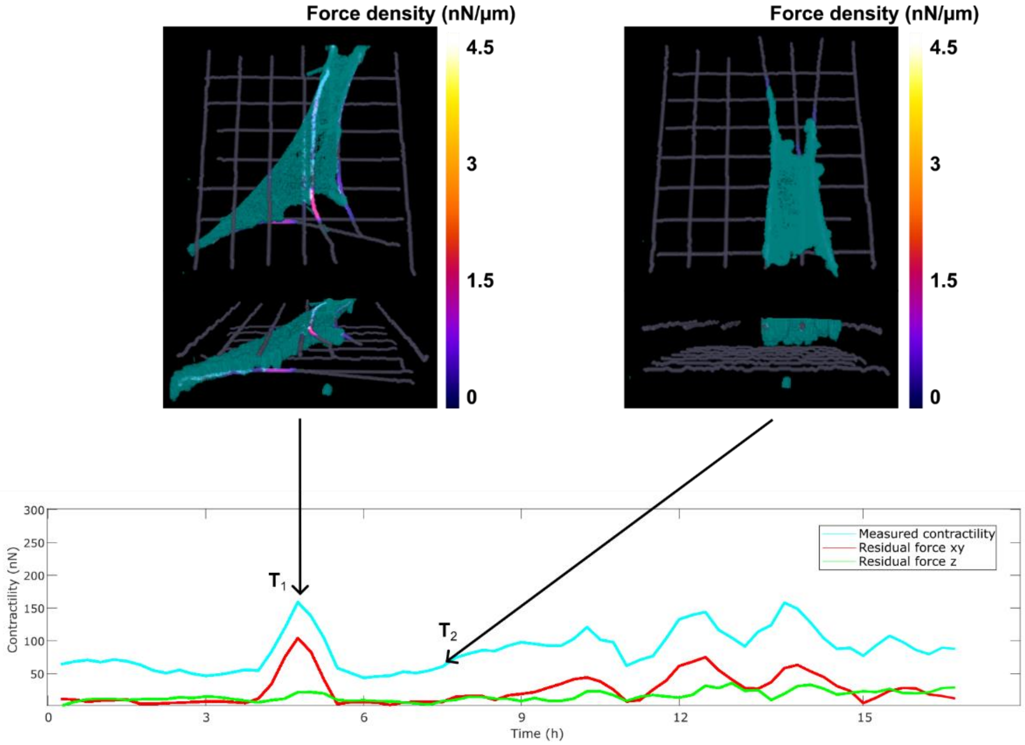

Supplementary Figure N3.1 - Residual forces along x-y and along z

Quantification of the x-y and z components of the residual forces for a HUVEC on a 2-layer fiber array with 10 µm lateral spacing and low-exposure fibers (T_{exp} = 600 µs, k = 3.5 nN/µm). The x-y component peaks at T_1, when the cell is spread on the two levels of fibers. By contrast, it remains close to zero at T_2, when the cell is only spread on the top layer.

Supplementary Note 4 – Experimental validation of the mechanical model

Experimental validation

To further validate our mechanical model of photopolymerized fibers as clamped beams under tensile stress, we selected a specific fiber condition (T_{exp} = 800 µs) and applied AFM tipless cantilever deflection at four distinct positions along the 80 µm-fiber: L/10, 2L/10, 3L/10, and 4L/10 (Sup.Fig. N4.1A,B). The resulting fiber deflection profiles provide insights into the dominant mechanical regime, distinguishing between structural stiffness and tension-driven behavior. Additionally, we tested the fiber response under higher forces (up to 15 nN), approaching the range applied by cells in our experiments, to exclude inelastic fiber behavior in this range of forces.

Results

The experimentally measured deflection profiles for forces of 1 nN, 5 nN, 10 nN, and 15 nN exhibit a linear relationship between distance from the edge and deflection, consistent with a tension-dominated mechanical regime (Sup. Fig. N4.1C).
Across all tested positions, the deflection scaled proportionally with force, supporting the assumption that the fiber remains in the linear elastic regime (Sup. Fig. N4.1D). Moreover, our experimental data aligns both qualitatively and quantitatively with the Finite Element mechanical model presented in our study, further validating our theoretical approach.

Limitations of the measurement

- **Large forces:**
  Applying forces beyond 15 nN at the fiber edges or 10 nN at the center led to significant fiber deflections. In these cases, fiber torsion was observed under brightfield microscopy, which altered the force curves and potentially introduced artifacts. Additionally, under extreme force conditions, the AFM cantilever may slide along the fiber instead of maintaining a fixed contact point, further complicating force measurements. This highlights a key limitation of using AFM at high forces: excessive deflections can induce mechanical instabilities such as twisting and sliding, which hinder quantitative analysis.

- **Contact area:**
  A key limitation of using a tipless cantilever is the uncertainty regarding the actual contact area between the cantilever and the fiber. Any minor tilt in the experimental setup or slight misalignment during fiber printing could shift the contact point from the cantilever center to its sides. In our validation experiment, we defined the contact point at the upper edge of the cantilever, in agreement with the expected infinite stiffness at the fiber edge, as shown in Sup. Fig. N4.1C. However, variations in contact geometry may introduce deviations in mechanical measurements.

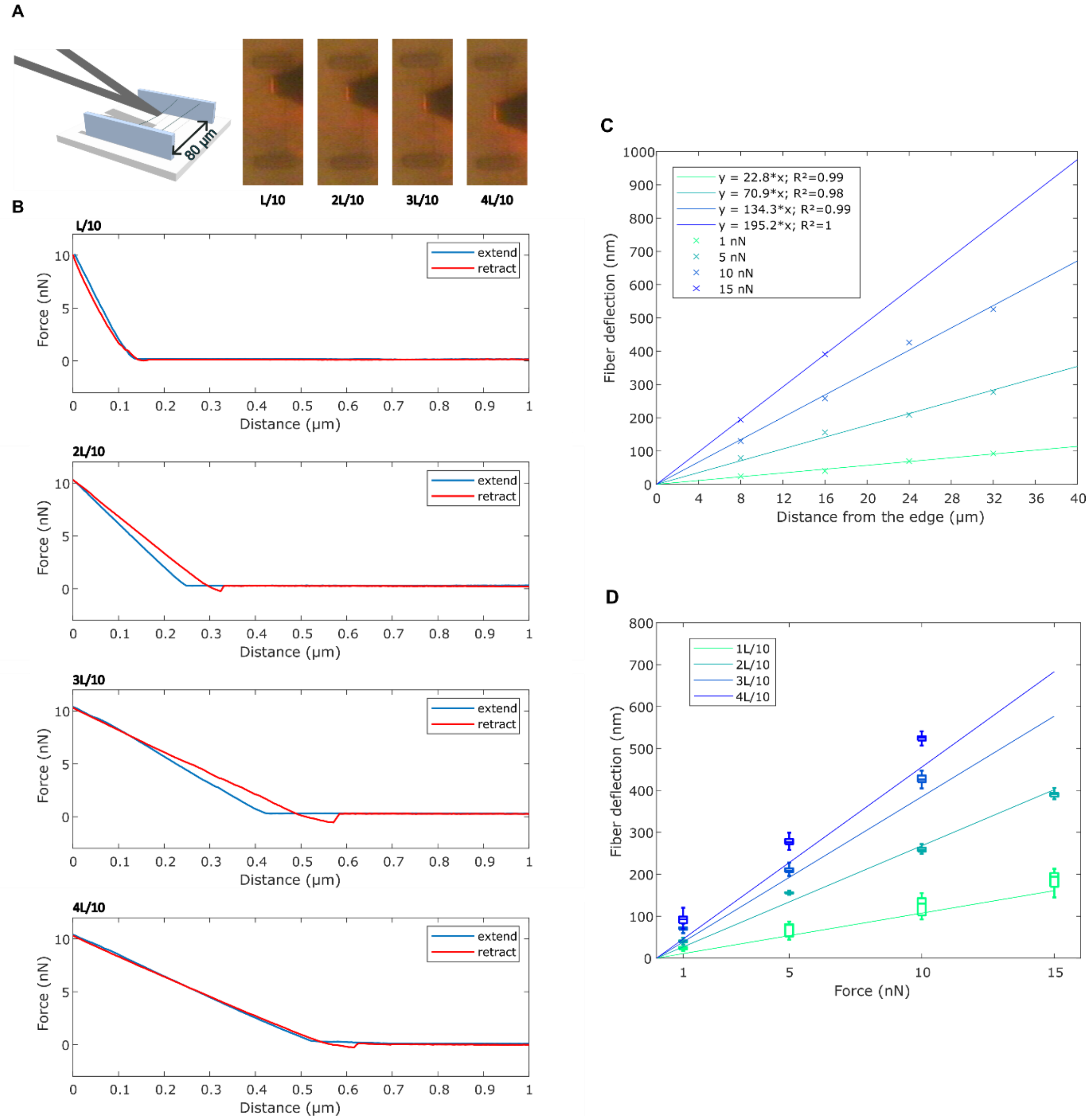

Supplementary Figure N4.1 – AFM fiber deflection profile

A. Position of the AFM cantilever respective to the photopolymerized fiber. **B.** Representative force-distance curves for an applied force of 10 nN at four different positions (L/10, 2L/10, 3L/10 and 4L/10). **C.** Evolution of the fiber deflection as a function of the distance from the fiber edge for high-stiffness fibers (T_{exp} = 800 µs), and for applied forces of 1 nN, 5 nN, 10 nN and 15 nN. Individual dots represent the median value of each tested condition (boxplots shown in D). **D.** Evolution of the fiber deflection as a function of the applied force at four different positions (L/10, 2L/10, 3L/10 and 4L/10; $1 \leq N \leq 3$ fibers and $n > 256$ analyzed force curves for each. The number of samples varies across force conditions and positions. At 1 nN, N = 1 fiber for each position (L/10, 2L/10, 3L/10, and 4L/10). At 5 nN, N = 3 fibers at L/10, N = 2 at 3L/10, and N = 1 at 2L/10 and 4L/10. At 10 nN, N = 3 fibers at L/10 and N = 1 at other positions (2L/10, 3L/10, and 4L/10). At 15 nN, N = 1 fiber at L/10 and 2L/10. Straight lines represent the predicted deflection using the FE model and fiber parameters described in our study. For all box plots, the central mark indicates the median, the edges of the box denote the 25th and 75th percentiles, the whiskers extend to the most extreme data points not considered outliers.

Supplementary Note 5 – MATLAB module for fiber deflection calculation

The computation of the 3D deflection vector of each fiber was performed using the ***FunCalculateDeflection*** MATLAB module developed for the study. The main steps of this module are as follows:

- The module takes as input a 3D image containing the segmented fibers, appearing as labelled voxels. Segmentation and dynamic tracking of the fibers from the original image has been performed prior to the deflection vector computation using the ***FunSegFibers3D***, ***FunTrackingFibers3D*** and ***Calculate_Intersection*** modules (Sup. Fig. N5.1A,B).

- Each individual fiber is treated separately, and skeletonized using the MATLAB ***bwskel*** function. The two extremities of the skeletonized voxel line are detected and their 3D coordinates are stored (Sup. Fig. N5.1C).

- The reference stress-free state of the fiber is computed as the path that minimizes the euclidean distance between the two extremities. This is done using the ***bwdist*** MATLAB function, summing the distance maps of the two extremities and finally using the ***imregionalmin*** MATLAB function (Sup. Fig. N5.1D,E).

- Finally, the 3D coordinates vectors of the deflected line and reference stress-free line are subsampled to the number of nodes chosen for the Finite Element Modeling formulation (e.g. Nb_nodes = 81) using linear interpolation. In the case of fibers oriented along the x axis, the deflection at node k is computed as the 1D euclidean distance between the y and z coordinates of the deflected line and reference stress-free line:
  $\Delta_{k,y} = |y_{k,deflected} - y_{k,reference}|$ and $\Delta_{k,z} = |z_{k,deflected} - z_{k,reference}|$

  Respectively, in the case of fibers oriented along the y direction, the deflection at node k is computed as the 1D euclidean distance between the x and z coordinates of the deflected line and reference stress-free line:
  $\Delta_{k,x} = |x_{k,deflected} - x_{k,reference}|$ and $\Delta_{k,z} = |z_{k,deflected} - z_{k,reference}|$
  (Sup. Fig. N5.1F).

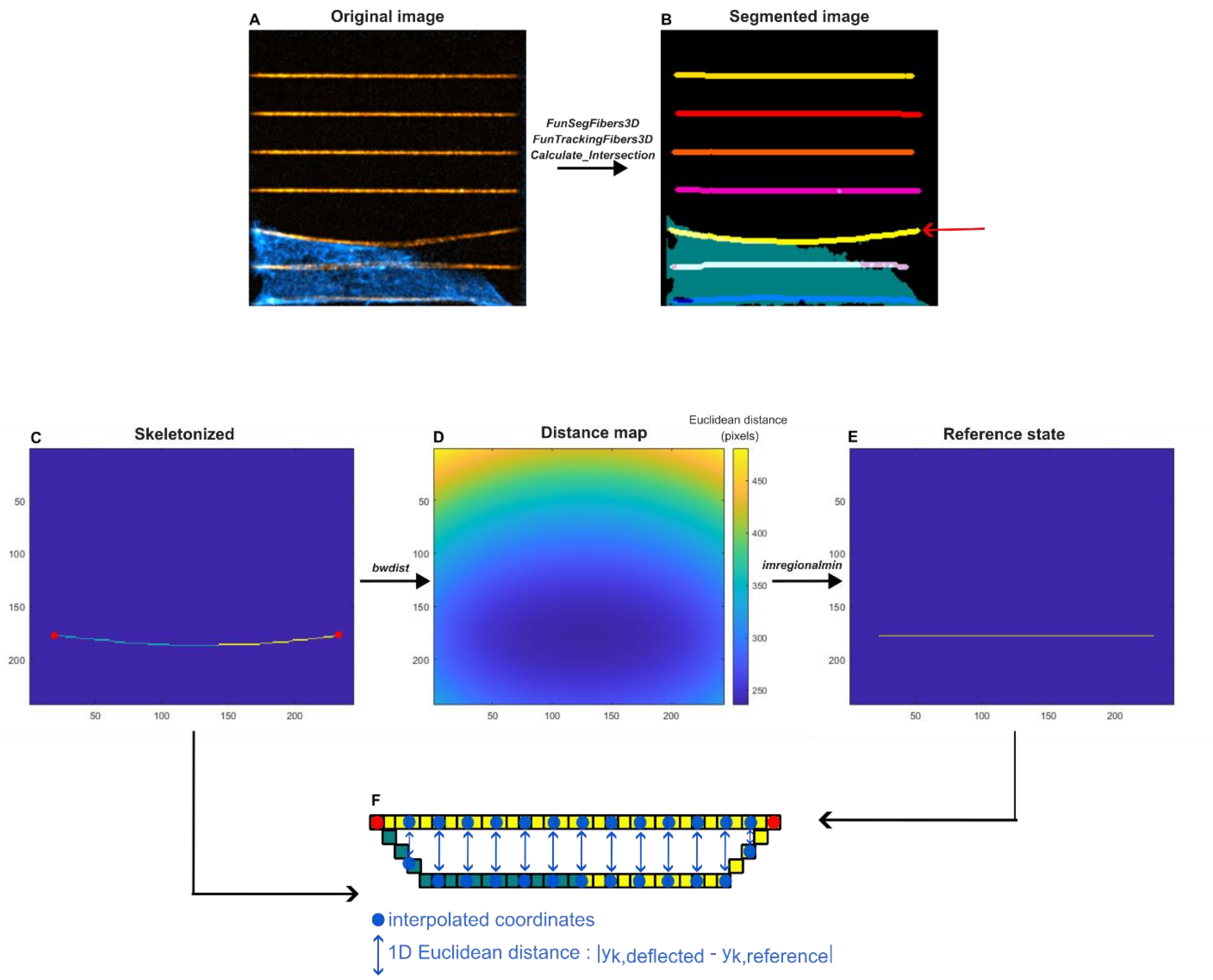

Supplementary Figure N5.1 – Deflection computation module

A. z-projection over one fiber layer of a 3T3 fibroblast deflecting photopolymerized fibers. **B.** z-projection of the segmented fibers and binary mask of the cell. **C.** 2D projection of the 3D skeletonized fiber corresponding to the fiber marked with a red arrow in **B**. The blue section of the fiber corresponds to the intersection area with the cell binary mask. The red dots mark the fiber extremities. **D.** 2D projection of the 3D distance map corresponding to the summation of the Euclidean distance from both fiber extremities. **E.** Resulting reference state computed as the regional minima of the distance map shown in **D**. **F.** Subsampling of the fiber 3D coordinates vector and computation of the voxel to voxel distance along the two lateral directions.

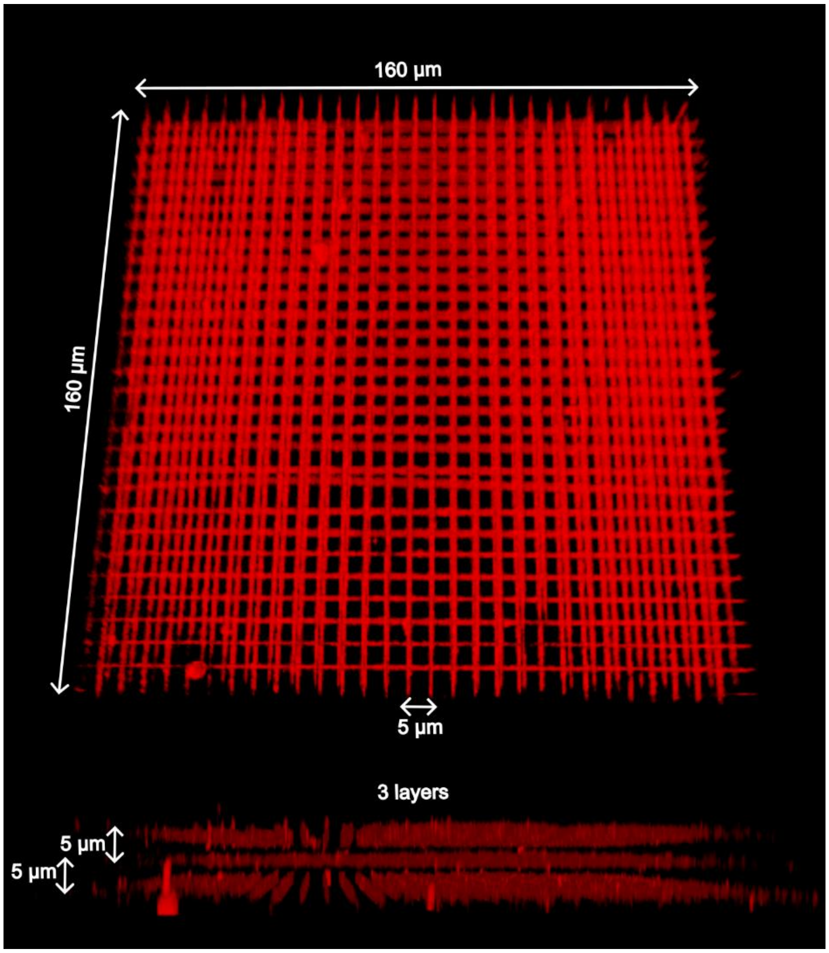

Supplementary Figure 1 – Multilayer 3D scaffolds

3D view of a 3-layer 3D microscaffold with both lateral spacing and interlayer spacing set to 5 µm (top: top view, bottom: side view; red: fluorescent fibronectin coated fibers, spinning disk imaging).

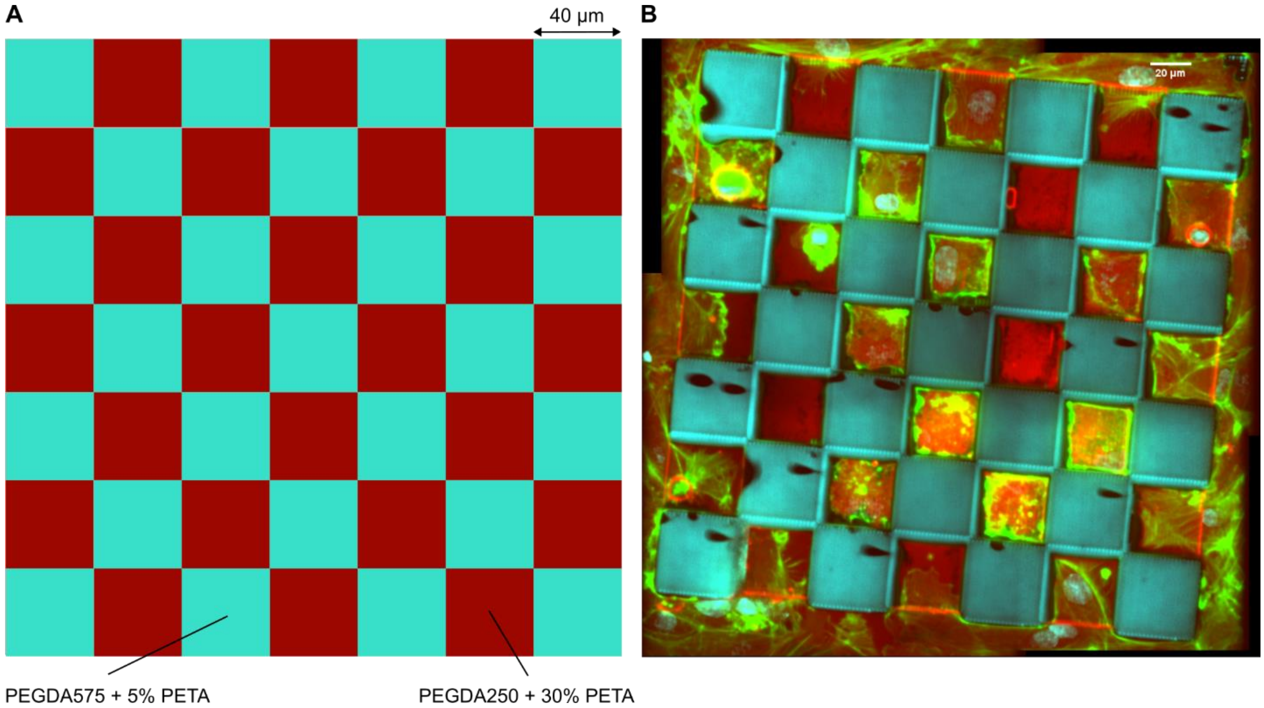

Supplementary Figure 2 - Spatially directed adhesion in composite microstructures

A. Schematic of a composite checkerboard 2D microstructure used to evaluate the choice of materials used to direct protein adsorption and cell adhesion. **B.** Actin (green) and nuclei (blue) staining of HUVEC cells seeded at high density on a fluorescent fibronectin (red) coated composite checkerboard and fixed after two hours. Cells exclusively adhere to the PEGDA250 part and adopt a square shape.

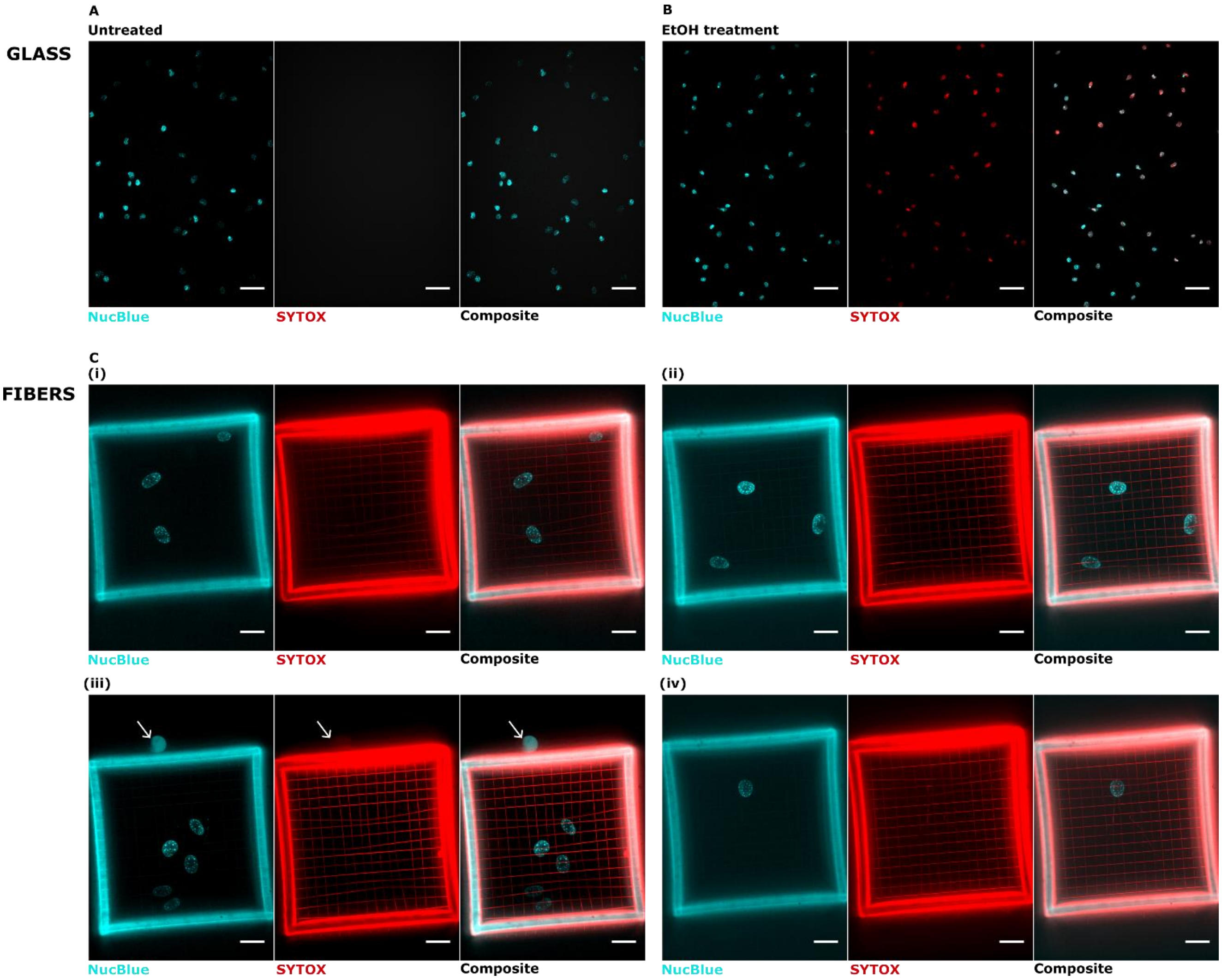

Supplementary Figure 3 – Cell viability on photopolymerized fiber scaffolds

Control experiments for the Live/Dead assay were conducted by seeding NIH/3T3 fibroblasts on glass under two conditions: **A.** untreated and **B.** exposed briefly to ethanol. In the ethanol-treated condition, strong SYTOX fluorescence colocalizing with NucBlue indicated compromised plasma membranes and cell death (scale bar = 50 µm). **C.** Representative images (i–iv) show NIH/3T3 fibroblasts cultured for 24 hours within photopolymerized fiber scaffolds. Of the cells imaged ($N_{scaffolds} = 16$, $N_{cells} = 32$), all remained viable except for one located at the scaffold periphery (iii, indicated by a white arrow). Note that SYTOX dye bound nonspecifically to the photopolymerized resin, resulting in fluorescence from the scaffold and fibers (scale bar = 25 µm).

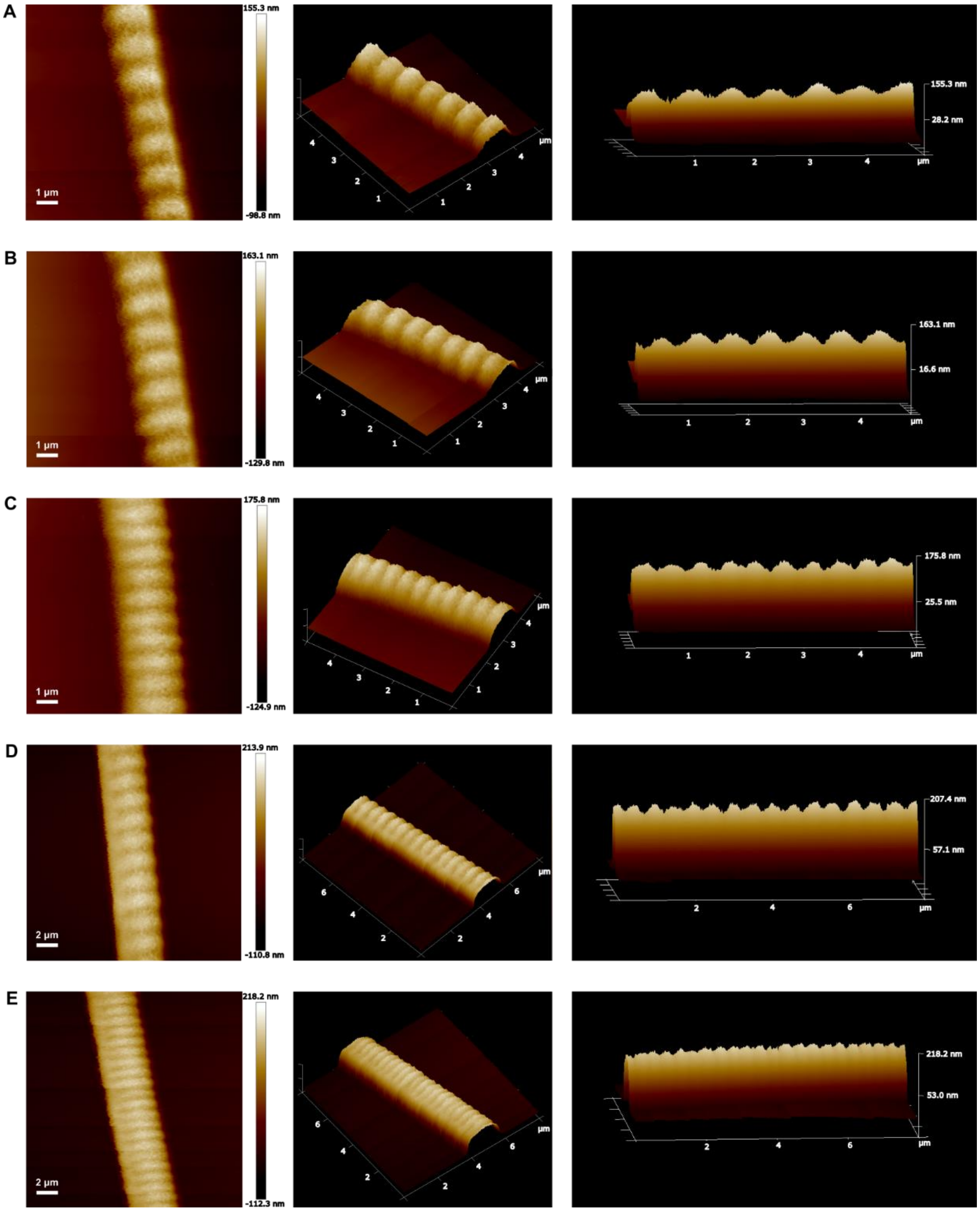

Supplementary Figure 4 - AFM topography scanning of the photopolymerized fibers

Topography images (left) and 3D views (middle: top view, right: side view) of the fibers for **A.** T_{exp} = 600 µs, **B.** T_{exp} = 650 µs, **C.** T_{exp} = 700 µs, **D.** T_{exp} = 750 µs and **E.** T_{exp} = 800 µs.

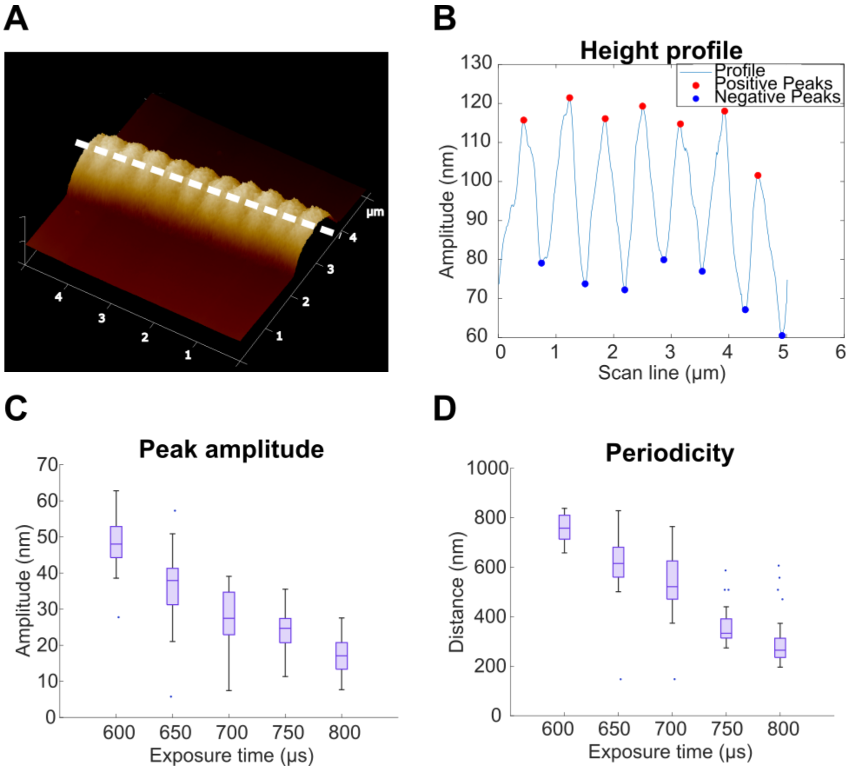

Supplementary Figure 5 - Nanostructuration

A. 3D view of a fiber and scan line (dashed white line). **B.** Height profile at the center of a fiber and positive and negative peak detection. **C.** Amplitude between successive positive and negative peaks for a range of exposure times. **D.** Nanostructuration periodicity (distance between successive positive peaks or successive negative peaks; N = 3 analyzed fibers for each condition). For all box plots, the central mark indicates the median, the edges of the box denote the 25th and 75th percentiles, the whiskers extend to the most extreme data points not considered outliers, and outliers are plotted individually (dots).

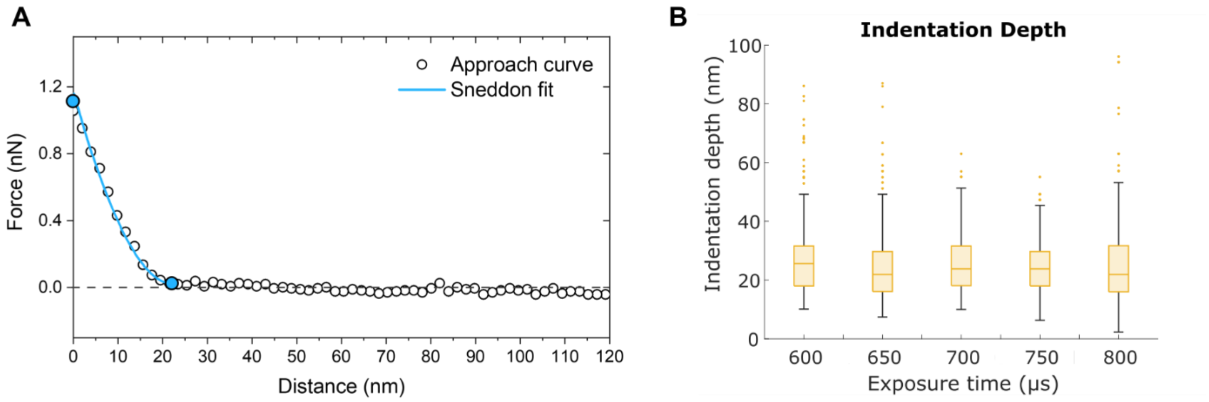

Supplementary Figure 6 - Indentation depth and force-distance curve

A. Typical force-distance curve obtained for the indentation experiment, here for T_{exp} = 700 μs and an applied load of 1 nN. **B.** Contact point for an applied load of 1 nN. For all box plots, the central mark indicates the median, the edges of the box denote the 25th and 75th percentiles, the whiskers extend to the most extreme data points not considered outliers, and outliers are plotted individually (dots).

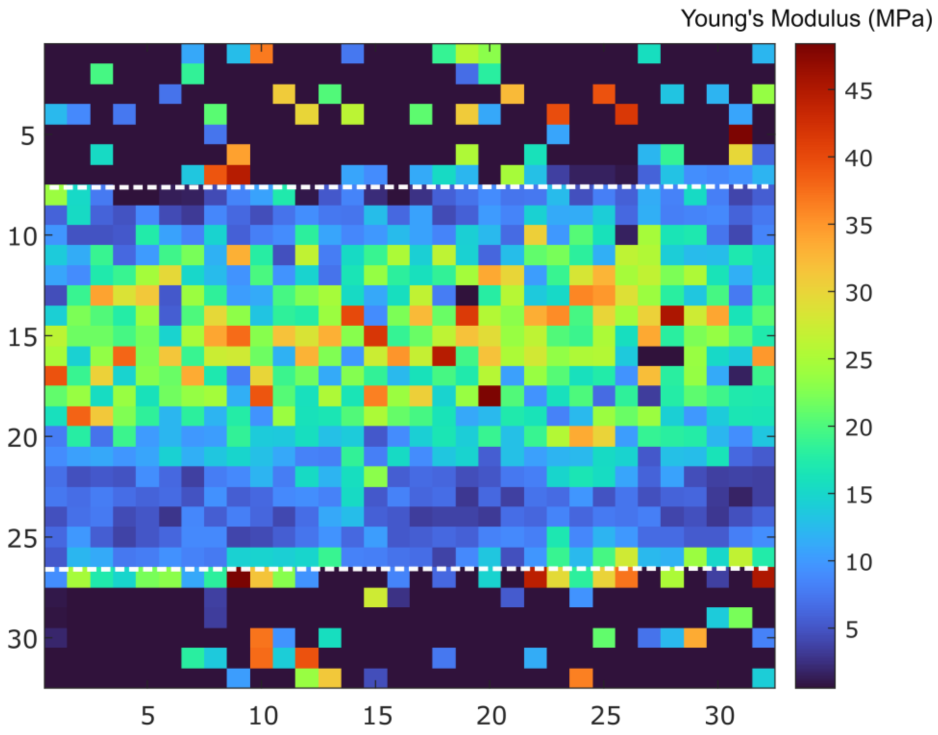

Supplementary Figure 7 – High-resolution indentation mapping

To better understand the high dispersion observed in the measured Young's moduli of photopolymerized fibers (Fig.2F), we performed high-resolution indentation mapping (32 × 32 force curves with ~150 nm pixel size, shown here for T_{exp} = 800 µs). Regions with Young's moduli exceeding 55 MPa, shown in black, correspond to the surrounding glass substrate, while the fiber lies within the white dotted outline. The map reveals a gradient in mechanical properties from the edges toward the fiber center, which likely contributes to the variability observed in individual modulus measurements.

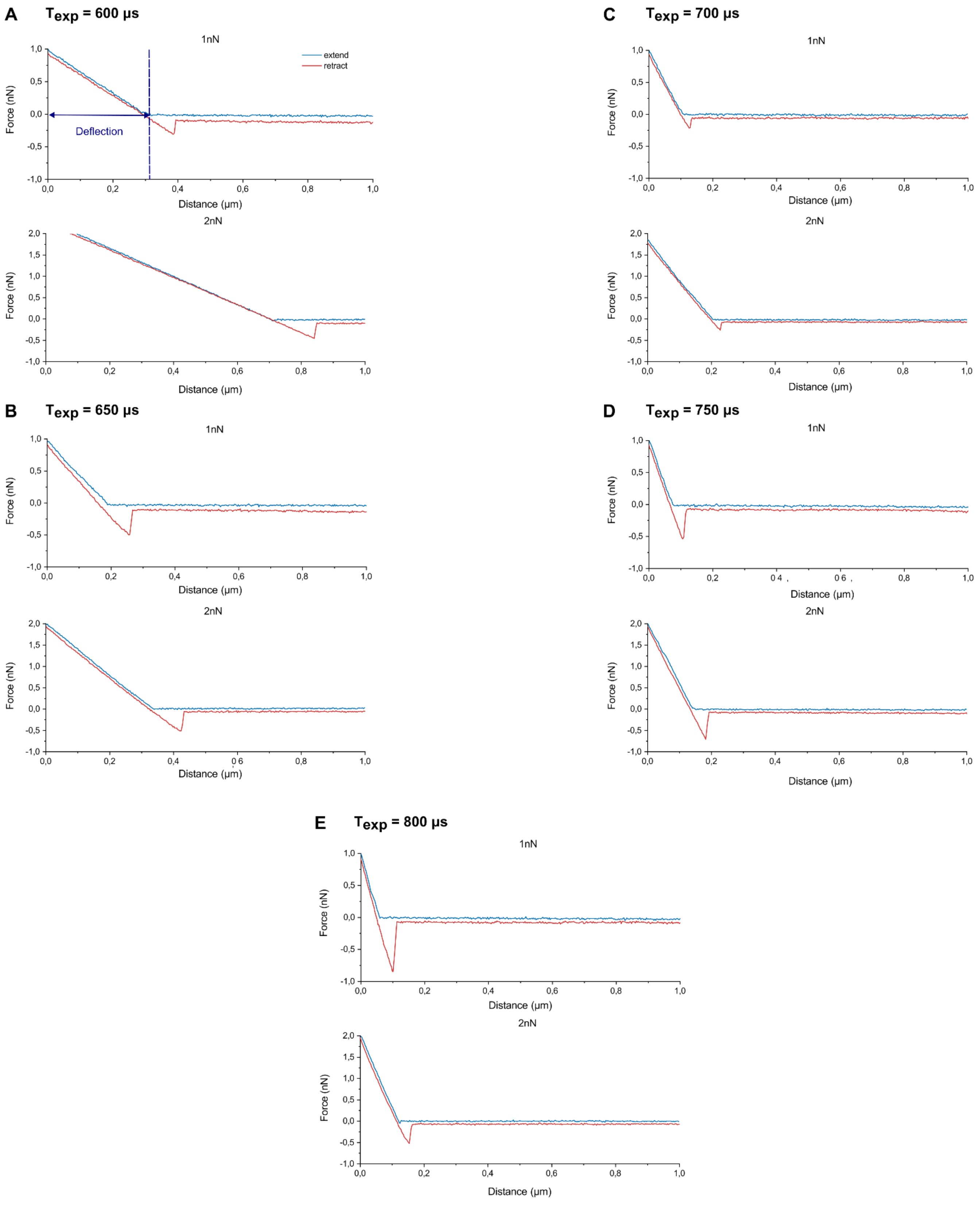
A
T_{exp} = 600 µs
1nN
extend
retract
Deflection
Force (nN)
Distance (µm)
2nN
C
T_{exp} = 700 µs
B
T_{exp} = 650 µs
D
T_{exp} = 750 µs
E
T_{exp} = 800 µs

Supplementary Figure 8 - Suspended fiber deflection experiment: force-distance curves

Typical force-distance curves obtained when deflecting a fiber with a tipless AFM cantilever for **A.** T_{exp} = 600 µs, **B.** T_{exp} = 650 µs, **C.** T_{exp} = 700 µs, **D.** T_{exp} = 750 µs and **E.** T_{exp} = 800 µs (applied load of top: 1 nN, bottom: 2 nN).

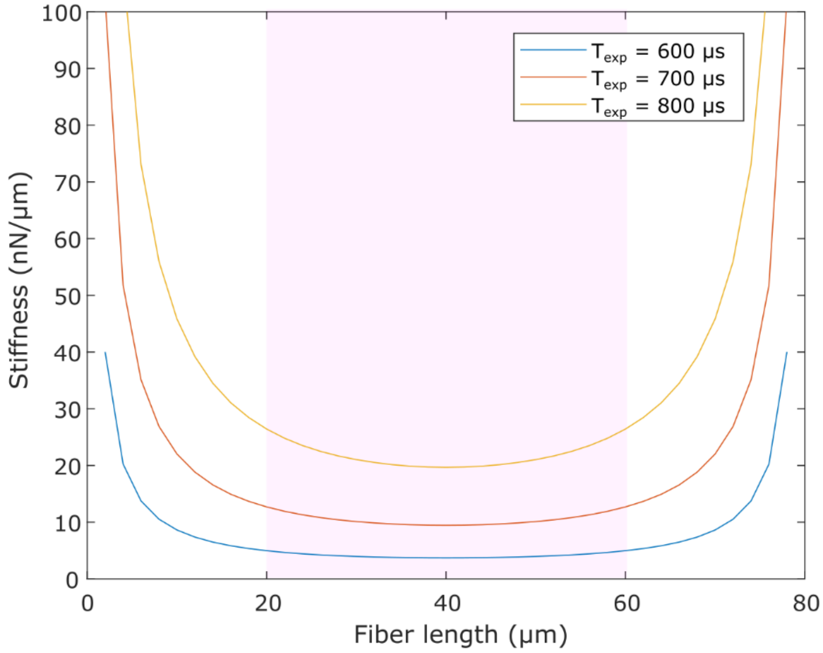

Supplementary Figure 9 - Stiffness profile of a clamped beam under tension

Theoretical stiffness profiles for photopolymerized fibers with T_{exp} = 600 µs, 700 µs or 800 µs. The shaded area depicts the central portion of the fiber used for average force per node calculation, where the stiffness does not exceed a 30% increase compared to the stiffness at the midpoint of the fiber.

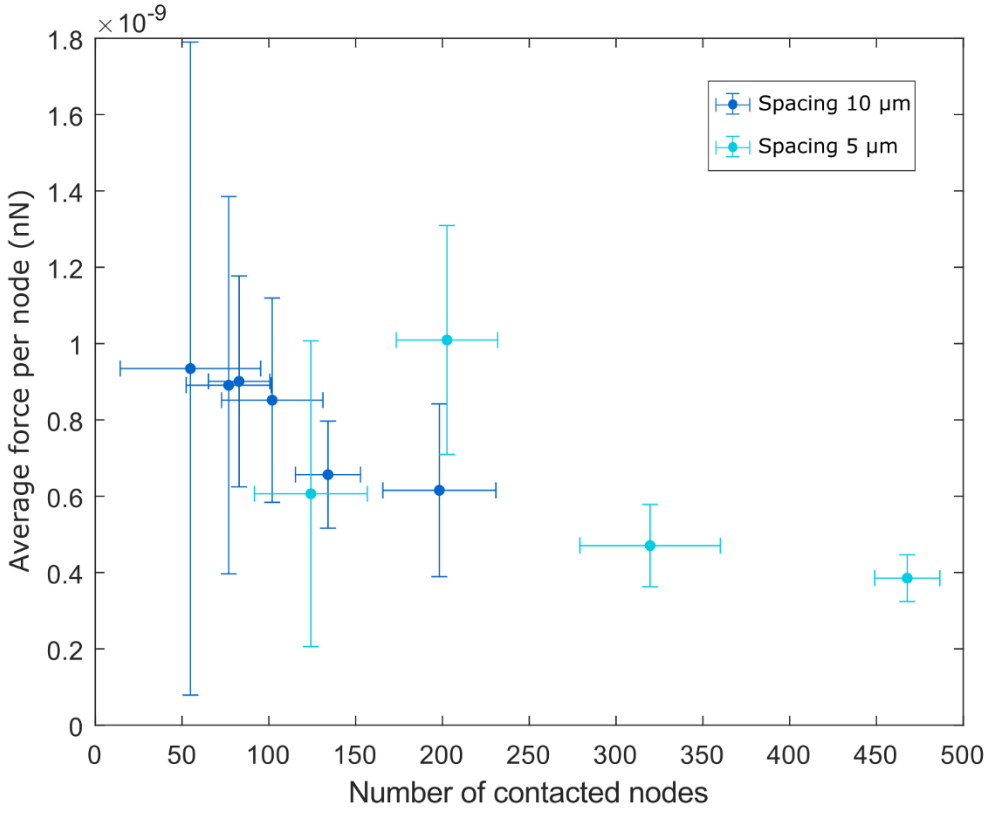

Supplementary Figure 10 - Average force per node decreases with the number of contacted nodes

Average force per node as a function of the number of contacted nodes for HUVECs on low-exposure fibers (T_{exp} = 600 µs, k = 3.5 nN/µm) with lateral spacing of 5 µm and 10 µm. Each point represents the mean value over the whole timelapse for an individual cell and error bars correspond to the standard deviation.

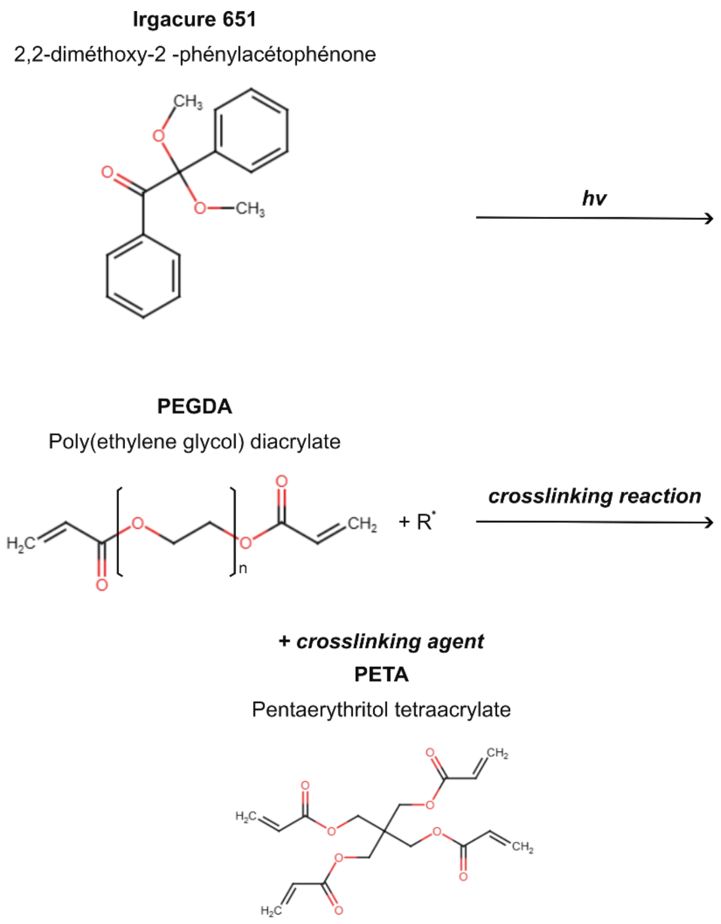

Supplementary Figure 11 – Photopolymerization reaction

Radical photopolymerization of PEGDA initiated by Irgacure 651 under UV light. Upon irradiation, Irgacure 651 undergoes homolytic cleavage to generate benzoyl and α-alkoxy radicals, which initiate acrylate polymerization and network crosslinking.

Supplementary Table

Supplementary Table 1 - Scanning speed

**Exposure Time (µs)**	**Scanning speed (µm/s)**
600	55.6
650	51.4
700	47.8
750	44.6
800	41.9

Materials and methods

Cell experiments

NIH/3T3 cells, ATCC CRL-1658, were transfected by PEI MAX (Polysciences) with Lifeact-GFP plasmid (pLVX LifeAct-eGFP-P2A-puro, constructed from pLVX puro vector Clontech Catalog No. 632164), selected with 1 µg/ml puromycin and sorted by FACS. NIH/3T3 Lifeact-GFP cells were maintained in DMEM + 10% calf serum (ATCC 61965-026) + 1 µg/ml puromycin, at 37°C, 5% CO_2.
HUVECs were prepared from human umbilical cords provided by AP-HP, Hôpital Saint-Louis, Unité de Thérapie Cellulaire, CRB-Banque de Sang de Cordon, Paris, France. HUVEC Lifeact-GFP cells were obtained from these cells by transduction with rLVUbi–LifeAct®–TagGFP2 (Ibidi, Gräfelfing, Germany). After thawing, cells were maintained for 1–2 passages in endothelial cell growth medium 2 (ECGM2, Promocell GmbH, Heidelberg, Germany) with 1% P-S, at 37 °C and 5% CO_2, in collagen-coated flasks. Measurements on HUVECs shown in Fig.4 were acquired from a single seeding experiment (n_seeding = 1), and are representative of the method's capability.
Dendritic cells were obtained as previously described[2] by differentiating bone-marrow cells from LifeAct-GFP mice during 10-11 days in IMDM medium (Sigma-Aldrich, Darmstadt, Germany) containing 10% FBS decomplemented and filtered (Biowest, Nuaillé, France), 20 mM L-glutamine (Gibco, Waltham, Massachusetts, USA), 100 U/ml penicillin–streptomycin (Gibco, Waltham, Massachusetts, USA), 50 µM 2-mercaptoethanol (Gibco, Waltham, Massachusetts, USA), and 50 ng/ml of GM-CSF containing supernatant obtained from transfected J558 cells[3]. Only semi adherent (dendritic cells) were recovered at the end of the culture (excluding non-adherent granulocytes and adherent macrophages). **Macrophages** were obtained by differentiating bone marrow cells from LifeAct-GFP mice in RMPI-Glutamax (Gibco) containing 10% FBS, 1% Pen/Strep, 10 mM Hepes, 1mM NaPyruvate, 50uM B-mercaptoethanol, and supplemented with 30% of M-CSF-containing L929 cell supernatant. Cells were let to differentiate for 7 days before removing M-CSF, and were used on day 8 or 9 after detachment with Trypsin.
Live/Dead assay was performed by culturing NIH/3T3 cells on fiber scaffolds for 24 hours, and incubating them for 30 minutes with 1 µL/mL SYTOX™ Deep Red Nucleic Acid Stain (Thermofisher) and 2 drops / mL NucBlue™ Live ReadyProbes™ Reagent (Hoechst 33342, Thermofisher) before imaging.

Imaging – Spinning disk

Live imaging experiments were performed using a spinning disk from the PICT-IBiSA platform, equipped with an inverted Eclipse Ti-E (Nikon, Tokyo, Japan) and a spinning disk CSU-X1 (Yokogawa) integrated in Metamorph software by Gataca Systems, with a Prime BSI camera (Photometrics, Tucson, AL, USA) with 6.5 µm pixel size and a z-motor Nanoz100 (Mad City Lab, Madison, WI, USA), temperature and CO_2 controllers from Life Imaging Systems, and with a Plan fluor 40X oil (NA 1.3) objective. Image stack size was 180x280x21 µm with a voxel size of 0.16 x 0.16 x 1 µm. For imaging HUVECs and NIH/3T3

cells, a time step of ΔT = 15 min was employed for a total imaging time of 15-17 hours, whereas for macrophages, it was set to ΔT = 3 min and the total imaging time was 2 hours.

Lattice Light Sheet imaging

Image acquisition was performed on a commercialized version of a previously described setup from 3i (Denver, USA). Cells were scanned incrementally through a 20 µm long light sheet, in 1000 nm steps using a fast piezoelectric flexure stage (equivalent to ≃ 650 nm voxel size in z axis after image realignment, respecting the 32,8° angle for the detection objective position). Data were imaged using sCMOS camera (Orca-Flash 4.0; Hamamatsu, Bridgewater, NJ). Excitation was achieved with 561 nm or 642 nm (MPB Communications, Montreal, Canada) diode lasers at ~5% acousto-optic tunable filter transmittance, with 50 mW nominal power. Excitation is done through a water-dipping 28.6x objective (NA=0.7, working distance 3.74-mm) and detection *via* a Nikon CFI Apo LWD 25x water-dipping objective (NA=1.1), completed with a 2.5x tube lens, to obtain a final pixel size of 104 x 104 nm. Lattice light-sheet imaging was performed using an excitation pattern of outer NA equal to 0.55 and inner NA equal to 0.493. Composite volumetric datasets were obtained using ≃ 10 ms exposure/optical planes/channel at a time resolution of 15 seconds per total cell volume of 80 planes with a field size of 1200x1200 pixels. Double labeled images were acquired on two separated cameras, using a dichroic mirror with an edge at 660 nm. 3D+time series were acquired within 20 minutes. Acquired data were deskewed, a necessary step to realigned the image frames, using LLSpy, a python library (copyright to T. Lambert, Harvard Medical School, Boston, USA; https://github.com/tlambert03/LLSpy) and deconvolved using cudaDeconv (copyright to Lin Shao et al, HHMI https://github.com/dmilkie/cudaDecon), included in LLSpy. For Lifeact-GFP cells on a two-layer microscaffold, LLSM images were denoised using ND-SAFIR software (https://gitlab.inria.fr/serpico/ndsafir_bin) before deskewing and deconvolution, as above. Napari, a multi-dimensional image viewer for Python (https://github.com/napari/napari).

3D segmentation and tracking of the fibers and deflection measurement

Pretreatment

Pretreatment of the acquired 3D timelapses was performed using the ImageJ software and consisted of drift correction using the 3D Drift Correction plug-in, resampling with bilinear interpolation to achieve a final isotropic voxel size of 0.3 µm and background subtraction. 3D segmentation, tracking, deflection calculation and force computation were carried out using a custom Matlab script.

3D segmentation and tracking

Briefly, the volumetric image was thresholded and the extremities of fibers were segmented in a semi-automated manner for the first time point. For each subsequent time point, the same threshold was applied to the image, and fibers were segmented as individual connected components. A lower volume

threshold was applied to filter small components, while an upper volume threshold was also set to exclude contacting fibers, which could occur in cases of strong deflections. These cases were treated separately, and the two connected fibers were split by computing the minimum geodesic distance between the two extremities of each fiber. Fiber tracking was performed by matching objects with the higher overlap between time points.

Deflection measurement

For each time point and each fiber, the local deflection amplitude was computed as the Euclidean distance between the skeletonized deflected fiber and the straight line connecting its two extremities of the fiber. This distance was evaluated separately along the y and z directions for each voxel, and the resulting vector was then subsampled to match the number of nodes used in the FEM model. A more detailed description of the deflection computation is given in Supplementary Note 5.

Finite Element Modeling

Each individual fiber was modeled as a rectangular beam under extensive stress and discretized into 3D beam elements, with N nodes of size l. The number of nodes was chosen as a trade-off between accuracy of the force reconstruction and computation time and was fixed at 41 (Supplementary Note 2). For experiments involving HUVECs, NIH-3T3 and Dendritic Cells, the node size was 2 µm while for Macrophages, it was 4 µm. Four degrees of freedom were considered: two transverse deflections, u_y and u_z, and two rotations, θ_y and θ_z, around the y and z axes. The total energy U_{tot} was calculated as the sum of the strain energy U_e and the potential energy related to the extensive stress U_n : $U_{tot} = U_e + U_n$. Using Castigliano's theorem, the stiffness matrix $[K]$ that links the applied stress $\{F\}$ to the resulting strain $\{\delta\}$ was decomposed into $[K_e]$ and $[K_n]$:

$$\{F\} = \nabla U_{tot} = \nabla(U_e + U_n) = [K]\{\delta\} = ([K_e + K_n])\{\delta\}$$

The resulting stiffness matrices were:

$$K_e = \frac{EI}{l^3}\begin{bmatrix} 12 & 0 & 0 & 6l & -12 & 0 & 0 & 6l \\ 0 & 12 & -6l & 0 & 0 & -12 & -6l & 0 \\ 0 & -6l & 4l^2 & 0 & 0 & 6l & 2l^2 & 0 \\ 6l & 0 & 0 & 4l^2 & -6l & 0 & 0 & 2l^2 \\ -12 & 0 & 0 & -6l & 12 & 0 & 0 & -6l \\ 0 & -12 & 6l & 0 & 0 & 12 & 6l & 0 \\ 0 & -6l & 2l^2 & 0 & 0 & 6l & 4l^2 & 0 \\ 6l & 0 & 0 & 2l^2 & -6l & 0 & 0 & 4l^2 \end{bmatrix}$$

and

$$K_n = \frac{P}{10l}\begin{bmatrix} 12 & 0 & 0 & l & -12 & 0 & 0 & l \\ 0 & 12 & -l & 0 & 0 & -12 & -l & 0 \\ 0 & -l & \frac{4l^2}{3} & 0 & 0 & l & -\frac{l^2}{3} & 0 \\ l & 0 & 0 & \frac{4l^2}{3} & -l & 0 & 0 & -\frac{l^2}{3} \\ -12 & 0 & 0 & -l & 12 & 0 & 0 & -l \\ 0 & -12 & l & 0 & 0 & 12 & l & 0 \\ 0 & -l & -\frac{l^2}{3} & 0 & 0 & l & \frac{4l^2}{3} & 0 \\ l & 0 & 0 & -\frac{l^2}{3} & -l & 0 & 0 & \frac{4l^2}{3} \end{bmatrix}$$

The detailed calculation of the stiffness matrices can be found in Supplementary Note 1.

Computational implementation of the FE model was performed by adapting the Matlab toolbox CALFEM (https://github.com/CALFEM/calfem-matlab). Specifically, the stiffness matrix provided in the "beam3e" function was replaced by the stiffness matrix described above.

References

1. Tanimoto, H. & Sano, M. A Simple Force-Motion Relation for Migrating Cells Revealed by Multipole Analysis of Traction Stress. *Biophys J* **106**, 16–25 (2014).
2. Faure-André, G. *et al.* Regulation of dendritic cell migration by CD74, the MHC class II-associated invariant chain. *Science* **322**, 1705–1710 (2008).
3. Gil-Torregrosa, B. C. *et al.* Control of cross-presentation during dendritic cell maturation. *European Journal of Immunology* **34**, 398–407 (2004).

List of supplementary movies

Supplementary movie S1: Time lapse representing a NIH/3T3 Lifeact-GFP cell on a two-layer microscaffold (L = 80 µm) with highly deformable low-exposure fibers. The cell colormap codes for the *z*-position of the cell (purple = lower plane; blue = upper plane). Red: fibers. Total duration is 15 hours. Spinning-disk microscopy.

Supplementary movie S2: Time lapse representing a HUVEC Lifeact-GFP cell on a two-layer microscaffold (L = 80 µm) with highly deformable low-exposure fibers. The cell colormap codes for the z-position of the cell (purple = lower plane; blue = upper plane). Red: fibers. Total duration is 15 hours. Spinning-disk microscopy.

Supplementary movie S3: Time lapse representing HUVECs Lifeact-GFP on a two-layer large microscaffold (L = 200 µm) with highly deformable low-exposure fibers. The cell colormap codes for the *z*-position of the cell (purple = lower plane; blue = upper plane). Red: fibers. Total duration is 8 hours. Spinning-disk microscopy.

Supplementary movie S4: Time lapse representing HUVECs Lifeact-GFP on a two-layer large microscaffold (L = 200 µm) with moderately deformable high-exposure fibers. The cell colormap codes for the *z*-position of the cell (purple = lower plane; blue = upper plane). Red: fibers. Total duration is 8 hours. Spinning-disk microscopy.

Supplementary movie S5: Time lapse imaging of the development step following fiber polymerization during the microfabrication process. At t = 4 s, the monomer solution is washed with an excess of ethanol 100% and tension builds up within seconds. Total duration is 10 seconds.

Supplementary movie S6: 3D deflection map (left) and traction force map (right) of a NIH/3T3 cell on low-exposure fibers (T_{exp} = 600 µs, k = 3.5 nN/µm, L = 80 µm, lateral spacing = 10 µm). For both maps, a maximum projection, top view and side view are represented. Bottom: evolution of contractility over time. Total duration is 15 hours.

Supplementary movie S7: 3D deflection map (left) and traction force map (right) of a NIH/3T3 cell on medium-exposure fibers (T_{exp} = 700 µs, k = 8.3 nN/µm, L = 80 µm, lateral spacing = 10 µm). For both maps, a maximum projection, top view and side view are represented. Bottom: evolution of contractility over time. Total duration is 15 hours.

Supplementary movie S8: 3D deflection map (left) and traction force map (right) of a NIH/3T3 cell on high-exposure fibers (T_{exp} = 800 µs, k = 17.6nN/µm, L = 80 µm, lateral spacing = 10 µm).

For both maps, a maximum projection, top view and side view are represented. Bottom: evolution of contractility over time. Total duration is 15 hours.

Supplementary movie S9: 3D deflection map (left) and traction force map (right) of a HUVEC cell on low-exposure fibers (T_{exp} = 600 µs, k = 3.5 nN/µm, L = 80 µm, lateral spacing = 10 µm). For both maps, a maximum projection, top view and side view are represented. Bottom: evolution of contractility over time. Total duration is 17 hours.

Supplementary movie S10: 3D deflection map (left) and traction force map (right) of a HUVEC cell on low-exposure fibers (T_{exp} = 600 µs, k = 3.5 nN/µm, L = 80 µm, lateral spacing = 5 µm). For both maps, a maximum projection, top view and side view are represented. Bottom: evolution of contractility over time. Total duration is 15 hours.

Supplementary movie S11: 3D deflection map (left) and traction force map (right) of a HUVEC cell on medium-exposure fibers (T_{exp} = 700 µs, k = 8.3 nN/µm, L = 80 µm, lateral spacing = 10 µm). For both maps, a maximum projection, top view and side view are represented. Bottom: evolution of contractility over time. Total duration is 9 hours.

Supplementary movie S12: 3D deflection map (left) and traction force map (right) of a HUVEC cell on high-exposure fibers (T_{exp} = 800 µs, k = 17.6 nN/µm, L = 80 µm, lateral spacing = 10 µm). For both maps, a maximum projection, top view and side view are represented. Bottom: evolution of contractility over time. Total duration is 17 hours.

Supplementary movie S13: 3D deflection map (left) and traction force map (right) of a macrophage on low-exposure fibers (T_{exp} = 600 µs, k = 1.8 nN/µm, L = 160 µm, lateral spacing = 5 µm). For both maps, a maximum projection, top view and side view are represented. Bottom: evolution of contractility over time. Total duration is 130 minutes.

Supplementary movie S14: 3D deflection map (left) and traction force map (right) of a macrophage on low-exposure fibers (T_{exp} = 600 µs, k = 1.8 nN/µm, L = 160 µm, lateral spacing = 10 µm). For both maps, a maximum projection, top view and side view are represented. Bottom: evolution of contractility over time. Total duration is 130 minutes.

Supplementary movie S15: 3D deflection map (left) and traction force map (right) of a macrophage on medium-exposure fibers (T_{exp} = 700 µs, k = 4.1 nN/µm, L = 160 µm, lateral spacing = 5 µm). For both maps, a maximum projection, top view and side view are represented. Bottom: evolution of contractility over time. Total duration is 130 minutes.

Supplementary movie S16: 3D deflection map (left) and traction force map (right) of a macrophage on high-exposure fibers (T_{exp} = 800 µs, k = 8.8 nN/µm, L = 160 µm, lateral spacing = 10 µm). For both maps, a maximum projection, top view and side view are represented. Bottom: evolution of contractility over time. Total duration is 130 minutes.

Supplementary movie S17: Time lapse representing a dendritic cell Lifeact-GFP cell on a two-layer microscaffold with low-exposure fibers (T_{exp} = 600 µs, k = 3.5 nN/µm, L = 80 µm, lateral spacing = 10 µm). Pink: F-actin, green: fibers. Total duration is 20 minutes. Lattice Light-Sheet microscopy.

Supplementary movie S18: 3D deflection map (left) and traction force map (right) of a dendritic cell on low-exposure fibers (T_{exp} = 600 µs, k = 3.5 nN/µm, L = 80 µm, lateral spacing = 10 µm), analysis of the Supplementary movie S17. For both maps, a maximum projection, top view and side view are represented. Bottom: evolution of contractility over time. Total duration is 20 minutes.
